# *Torvosaurus gurneyi* n. sp., the Largest Terrestrial Predator from Europe, and a Proposed Terminology of the Maxilla Anatomy in Nonavian Theropods

**DOI:** 10.1371/journal.pone.0088905

**Published:** 2014-03-05

**Authors:** Christophe Hendrickx, Octávio Mateus

**Affiliations:** 1 Departamento de Ciências da Terra, Faculdade de Ciências e Tecnologia, Universidade Nova de Lisboa, Caparica, Portugal; 2 Museu da Lourinhã, Lourinhã, Portugal; Monash University, Australia

## Abstract

The Lourinhã Formation (Kimmeridgian-Tithonian) of Central West Portugal is well known for its diversified dinosaur fauna similar to that of the Morrison Formation of North America; both areas share dinosaur taxa including the top predator *Torvosaurus*, reported in Portugal. The material assigned to the Portuguese *T. tanneri*, consisting of a right maxilla and an incomplete caudal centrum, was briefly described in the literature and a thorough description of these bones is here given for the first time. A comparison with material referred to *Torvosaurus tanneri* allows us to highlight some important differences justifying the creation of a distinct Eastern species. *Torvosaurus gurneyi* n. sp. displays two autapomorphies among Megalosauroidea, a maxilla possessing fewer than eleven teeth and an interdental wall nearly coincidental with the lateral wall of the maxillary body. In addition, it differs from *T. tanneri* by a reduced number of maxillary teeth, the absence of interdental plates terminating ventrally by broad V-shaped points and falling short relative to the lateral maxillary wall, and the absence of a protuberant ridge on the anterior part of the medial shelf, posterior to the anteromedial process. *T. gurneyi* is the largest theropod from the Lourinhã Formation of Portugal and the largest land predator discovered in Europe hitherto. This taxon supports the mechanism of vicariance that occurred in the Iberian Meseta during the Late Jurassic when the proto-Atlantic was already well formed. A fragment of maxilla from the Lourinhã Formation referred to *Torvosaurus* sp. is ascribed to this new species, and several other bones, including a femur, a tibia and embryonic material all from the Kimmeridgian-Tithonian of Portugal, are tentatively assigned to *T. gurneyi*. A standard terminology and notation of the theropod maxilla is also proposed and a record of the *Torvosaurus* material from Portugal is given.

## Introduction

The Upper Jurassic beds of central Portugal have yielded numerous dinosaur taxa representing one of the richest European faunas of dinosaurs from the Mesozoic, and certainly the most diverse one from the Late Jurassic of Europe. Members of all major clades of dinosaurs other than marginocephalians are represented, and theropods are by far the most diversified group of the clade Dinosauria [1]–[3]. Hitherto, tracks, eggs, teeth and bone material (including embryos and hatchlings) discovered in the Alcobaça Formation (Kimmeridgian) of the Guimarota mine [1] and Lourinhã Formation (Kimmeridgian-Tithonian) of the Lourinhã region [3], [4] have been assigned to at least ten theropod taxa belonging to the clade of Ceratosauridae [4], [5], Abelisauridae [6], Megalosauridae [4], [6]–[9], Allosauroidea [4], [10]–[15], Tyrannosauroidea [16], Compsognathidae [17], Avialae [18], [19], and some uncertain systematic theropod clades [6], [17], [20].

The Alcobaça and Lourinhã Formation are comparable to the contemporaneous Morrison Formation of Northern America both paleoenvironmentally and sedimentologically [3]. Most of non-coelurosaurian taxa (i.e., *Allosaurus*, *Ceratosaurus* and *Torvosaurus*) were present on both continents, indicating some faunal exchanges between the Iberian Meseta and North America in the Late Jurassic, although an intercontinental sea was already separating them [3], [21]. Mateus et al. [21] proposed that during the Callovian/Oxfordian transition, there were temporary land bridges that allowed terrestrial faunal exchange between North America and the Iberian Meseta. The high diversity of theropods in the Late Jurassic of Laurasia, represented by small, medium-sized and large individuals, indicates important niche partitioning between these carnivorous dinosaurs. The top predators at the acme of the food chain were represented by three large theropods, *Lourinhanosaurus*, *Ceratosaurus* and *Allosaurus*, and a very large form, *Torvosaurus*, functionally and ecologically similar to the super-predators *Carcharodontosaurus* and *Tyrannosaurus* from the Late Cretaceous of Africa and North America, respectively.

*Torvosaurus* has been reported several times in the Upper Jurassic of central Portugal in the locality of Casal do Bicho (Alcobaça), Quinta do Gradil (Cadaval), Praia da Corva (Porto Novo) and Praia da Vermelha (Lourinhã). This taxon is represented by a large tibia (ML 430) and a left maxilla (ML 1100) briefly described by Mateus & Antunes [7] and Mateus et al. [4], respectively, as well as a distal end of a femur (ML 632), a caudal vertebra (ML 1100) and a fragment of an unidentified limb bone (ML 1100) reported by Mateus et al. [4]. Malafaia et al. [8] published a fragment of right maxilla (ALT–SHN.116) whereas a mesialmost tooth (ML 962) was described by Hendrickx & Mateus [6]. Finally, embryonic remains (ML1188) discovered among a clutch of eggs have recently been reported by Araújo et al. [9]. These elements were all ascribed to the genus *Torvosaurus* or the species *Torvosaurus tanneri* although differences have been noted between the material from Portugal and the United States [4].

The present work aims to propose a standard terminology of the maxilla for nonavian theropods as well as to provide a thorough description of the material ML 1100 assigned to the species *Torvosaurus tanneri* [4]. Attribution to this taxon will be discussed after a detailed comparison with other megalosaurid material. A review of the *Torvosaurus* material from Portugal will finally be given.

## Materials and Methods

### Institutional Abbreviations

See Text S1.

### Nomenclatural Act

The electronic edition of this article conforms to the requirements of the amended International Code of Zoological Nomenclature, and hence the new names contained herein are available under that Code from the electronic edition of this article. This published work and the nomenclatural acts it contains have been registered in ZooBank, the online registration system for the ICZN. The ZooBank LSIDs (Life Science Identifiers) can be resolved and the associated information viewed through any standard web browser by appending the LSID to the prefix “http://zoobank.org/”. The LSID for this publication is: urn:lsid:zoobank.org:pub:4BD514CF-2AF8-401E-AC21-CB703D08089B. The LSID for this publication is: urn:lsid:zoobank.org:act:189C1060-7887-4837-9E30-870079E2B2B9. The electronic edition of this work was published in a journal with an ISSN, and has been archived and is available from the following digital repositories: PubMed Central (http://www.ncbi.nlm.nih.gov/pmc) and LOCKSS (http://www.lockss.org).

### Proposed Terminology of the Maxilla Anatomy in Nonavian Theropods

**Figure 1:**
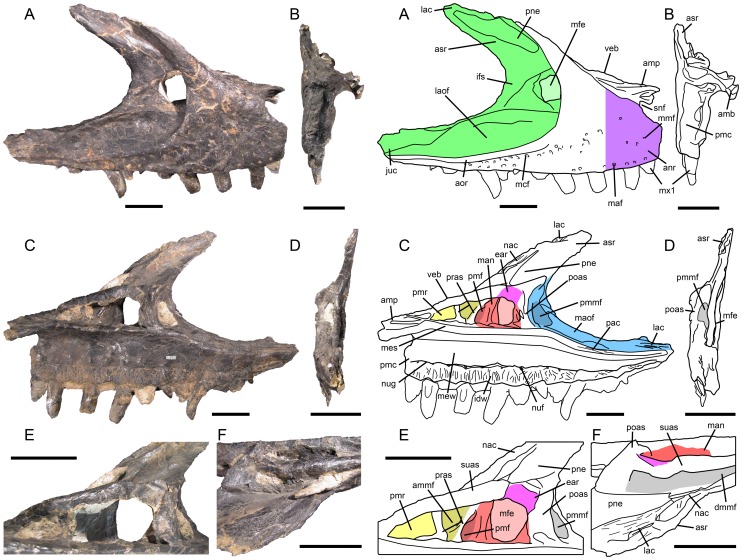
Proposed terminology and annotation of the nonavian theropod maxilla. Right maxilla of *Allosaurus fragilis* (USNM 8335) in **A**, lateral; **B**, anterior; **C**, medial and **D**, posterior views, with details of **E**, promaxillary recess and maxillary antrum in medial view; and **F**, ascending ramus and dorsal margin of vestibular bulla in dorsal view. Abbreviations: **ammf**, anteromedial maxillary fenestra; **amp**, anteromedial process; **anr**, anterior ramus; **aor**, antorbital ridge; **asr**, ascending ramus; **idw**, interdental wall; **ifs**, interfenestral strut; **juc**, jugal contact; **lac**, lacrimal contact; **laof**, lateral antorbital fossa; **law**, lateral wall; **maf**, maxillary alveolar foramina; **man**, maxillary antrum; **maof**, medial antorbital fossa; **mbo**, maxillary body; **mcf**, maxillary circumfenestra foramina; **mes**, medial shelf; **mew**, medial wall; **mfe**, maxillary fenestra; **mfo**, maxillary fossa; **mmf**, medial maxillary foramina; **mx1**, first maxillary tooth; **nac**, nasal contact; **nuf**, nutrient foramina; **nug**, nutrient groove; **pac**, palatine contact; **pmc**, premaxillary contact; **pmmf**, posteromedial maxillary fenestra; **pmr**, promaxillary recess; **pne**, pneumatic excavation; **poas**, postantral strut; **pras**, preantral strut; **snf**, subnarial foramen; **suas**, suprantral strut; **veb**, vestibular bulla. Scale bars = 5 cm.

**Figure 2:**
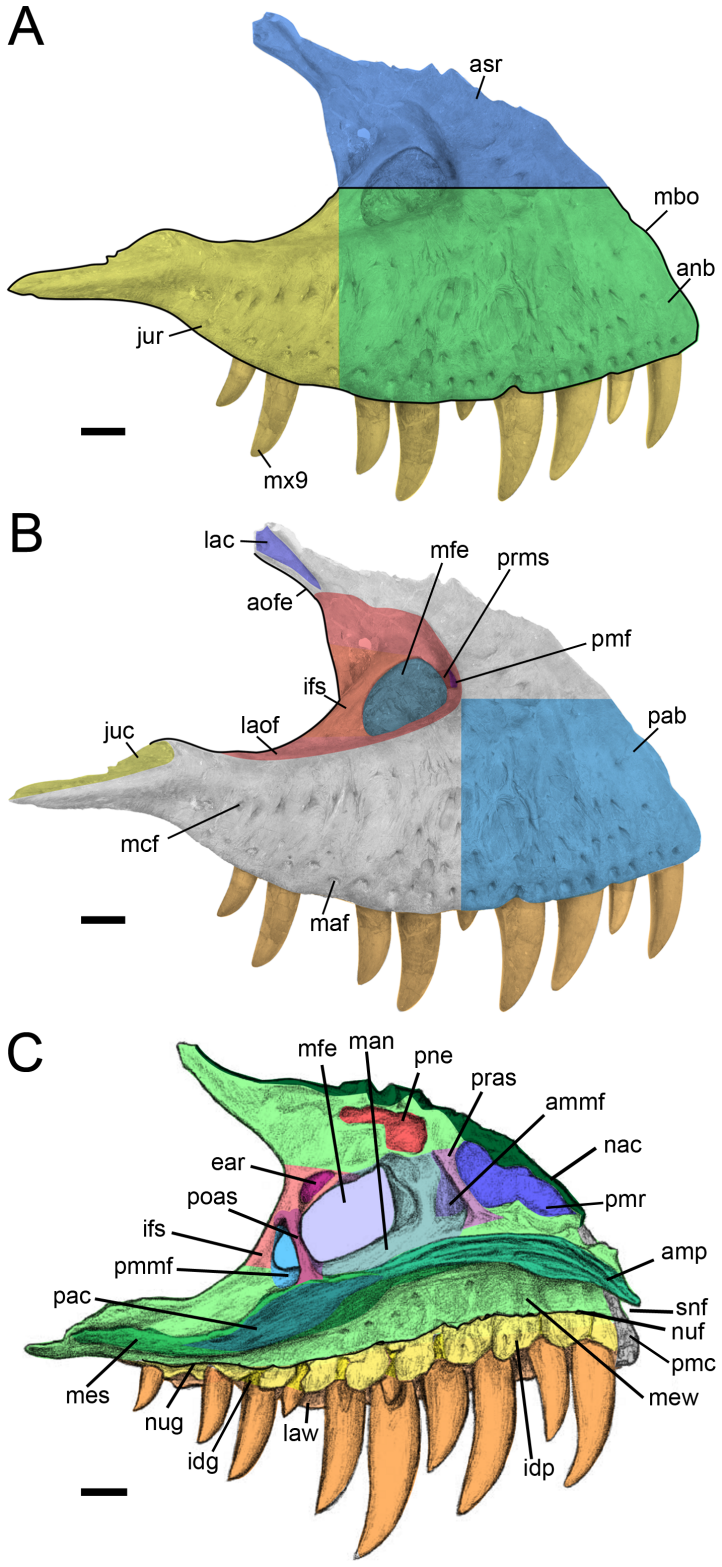
Proposed terminology and annotation of the nonavian theropod maxilla. Left maxillae of *Tyrannosaurus rex* in **A–B**, lateral view (CMNH 9380, reversed); and **C**, medial view (BHI 3033; modified from [32]). Abbreviations: **ammf**, anteromedial maxillary fenestra; **amp**, anteromedial process; **anb**, anterior body; **aofe**, antorbital fenestra; **asr**, ascending ramus; **ear**, epiantral recess; **idg**, interdental gap; **idp**, interdental plate; **ifs**, interfenestral strut; **juc**, jugal contact; **jur**, jugal ramus; **lac**, lacrimal contact; **laof**, lateral antorbital fossa; **law**, lateral wall; **maf**, maxillary alveolar foramina; **man**, maxillary antrum; **mbo**, maxillary body; **mcf**, maxillary circumfenestra foramina; **mes**, medial shelf; **mew**, medial wall; **mfe**, maxillary fenestra; **mx9**, ninth maxillary tooth; **nac**, nasal contact; **nuf**, nutrient foramina; **nug**, nutrient groove; **pab**, preantorbital body; **pac**, palatine contact; **pmc**, premaxillary contact; **pmf**, promaxillary fenestra; **pmmf**, posteromedial maxillary fenestra; **pmr**, promaxillary recess; **pne**, pneumatic excavation; **poas**, postantral strut; **pras**, preantral strut; **prms**, promaxillary strut; **snf**, subnarial foramen. Scale bars = 5 cm.

**Figure 3:**
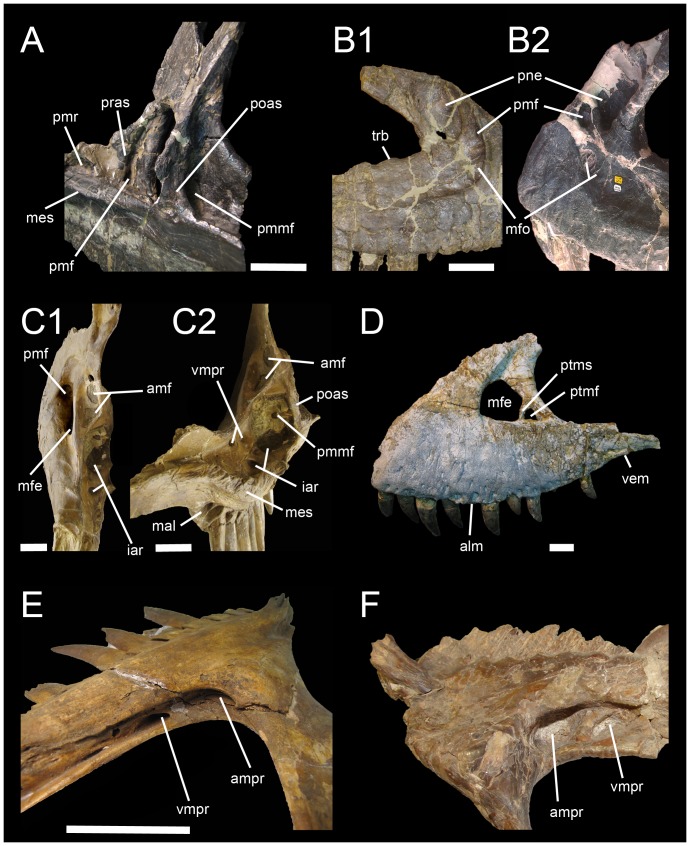
Proposed terminology and annotation of the nonavian theropod maxilla. **A**, Right maxilla of *Allosaurus fragilis* (AMNH 600) in posteromedial view; **B**, lateral antorbital fossae of *Ceratosaurus* in lateral view; **B1**, right maxilla of *Ceratosaurus magnicornis* (MWC 1) and; **B2**, left maxilla of *Ceratosaurus dentisulcatus* (UMNH VP 5278; courtesy of Roger Benson); **C**, left maxilla of *Tyrannosaurus rex* (CMNH 9380) in posterodorsal (**C1**) and dorsal (**C2**) views; **D**, left maxilla of *Tarbosaurus baatar* (ZPAL MgD-I/4; courtesy of Stephen Brusatte) in lateral view; **E**, right maxilla of *Duriavenator hesperis* (BMNH R332) in dorsomedial view; and **F**, left maxilla of *Piatnitzkysaurus floresi* (PVL 4073) in dorsomedial view (courtesy of Martin Ezcurra). Abbreviations: **amf**, accessory maxillary fenestra; **ammf**, anteromedial maxillary fenestra; **ampr** anteromedial pneumatic recess; **iar**, interalveolar recess; **mal**, maxillary alveoli; **mes**, medial shelf; **mfe**, maxillary fenestra; **mfo**, maxillary fossa; **pmf**, promaxillary fenestra; **pmmf**, posteromedial maxillary fenestra; **pmr**, promaxillary recess; **pne**, pneumatic recess; **poas**, postantral strut; **pras**, preantral strut; **ptmf**, postmaxillary fenestra; **ptms**, postmaxillary strut; **trb**, tooth root bulge; **vmpr**, ventromedial pneumatic recess. Scale bars = 5 cm.

The maxilla is a cranial bone displaying an important morphological variability among nonavian theropods (e.g., [9]:note 3; [22]:fig. 3; [23]:fig. 4.5). Such morphological variation shows the great taxonomical utility and systematic potential of the maxilla in this clade of dinosaurs. As this bone provides far more information than many other parts of the skeleton, and the diagnostic value of the maxilla is significant, particular attention should be accorded to the description of this bone in the literature on nonavian theropod anatomy. Nevertheless, the terminology and abbreviations of the maxilla anatomy have been inconsistent in nonavian theropods. Several different anatomical terms for the same maxilla sub-entity have been often used, as in some examples given below. An attempt of a standard terminology for the maxilla was already proposed by Witmer [24] who, however, mostly concentrated on the maxillary sinuses and did not provide a terminology for the maxillary ramus, processes and articulations. The present paper aims to propose a standardization of the anatomical terms for each of the maxilla sub-units (Figs. 1–3), mostly selected by their relevance, significance and importance in the theropod literature, in order to facilitate future description of this bone. The anatomical terms were grouped into nine sections, and each term is associated with a three to four letters abbreviation and followed by a definition. The nomenclature for pneumatic recesses and openings mostly follows the terminology given by Witmer [24] and only differs for a few terms. For clarity reasons, the internal antorbital fenestra, caudal fenestra of the maxillary antrum, and fenestra communicans of Witmer [24] are here referred to as the antorbital fenestra, posteromedial maxillary fenestra, and anteromedial maxillary fenestra, respectively. Gold et al. [25] noticed some confusion with the term “recess” in the literature and preferred using “promaxillary sinus” instead of “promaxillary recess”. Nevertheless, only one maxillary sinus may have invaded both maxillary antrum and promaxillary recess [26] and we therefore favoured Witmer's terminology. The presence of unnamed fossae/fenestrae within the antorbital fossa in some allosauroids (Fig. 1), tyrannosaurids (Figs. 2–3) and oviraptorosaurs have lead us to propose additional terms for several maxillary sub-units, namely: pneumatic fenestra, ventral maxillary fenestra, medial maxillary fenestra, dorsomedial maxillary fenestra, postmaxillary fenestra, anteromedial and posteromedial maxillary recesses, postmaxillary and preantral struts. Likewise, we are proposing the terms “interdental wall” for the continuous lamina formed by the fusion of interdental plates.

### Bodies, Rami and Processes

The anatomical term “ramus” was favoured over “process” for the large projecting parts of the maxilla (e.g., ascending ramus, jugal ramus, anterior ramus), the term “process” being referred to a smaller projection of bone (e.g., anteromedial process).

#### Maxillary body (mbo)

Ventral part of the maxilla that excludes the ascending ramus (Fig. 2A). The delimitation of the maxillary body from the ascending ramus is somewhat subjective. Usually, these two units are virtually delimited by a constriction formed by the antorbital fenestra and a concave step on the anterodorsal margin of the maxilla. However, the anterior margin of the maxillary body and the ascending ramus can be confluent. In that case, the maxillary body and the ascending ramus should be delimited by a virtual line starting from the apex of the curvature of the antorbital fenestra (which is not always the anteriormost point of the antorbital fenestra) and extending in parallel to the main axis of the ventral margin of the maxilla. The maxillary body, as used by several authors (e.g., [4], [27]–[31]), is also termed the main body (e.g., [32]–[37]). It includes two main anatomical units: the anterior body and the jugal ramus.

#### Anterior body (anb)

Anterior part of the maxillary body that extends from the premaxilla contact to the anteriormost point of the antorbital fenestra (Fig. 2A). The anterior body, corresponding to the ventral ramus of the nasal process of [38], includes both the preantorbital body and anterior ramus.

#### Preantorbital body (pab)

Anterior part of the maxillary body that extends from the premaxilla contact to the anteriormost point of the antorbital fossa (Fig. 2B). The preantorbital body, also known as the preantorbital process [29], is part of the anterior body.

#### Anterior ramus (anr)

Anterior projection of the maxillary body that extends from the premaxilla contact to a concave step on the anterodorsal margin of the maxilla that corresponds to the boundary between the maxillary body and the ascending ramus (Fig. 1A). The anterior ramus is considered to be absent when the anterodorsal margin of the maxillary body and the anterior margin of the ascending ramus are confluent. The anterior ramus, also called the rostral ramus [39] or anterior process (e.g., [37]–[42]) is part of the anterior body. It can also be part of the preantorbital body, or confluent with it when the concave step on the anterodorsal margin of the maxilla and the anteriormost point of the antorbital fossa are at the same level.

#### Ascending ramus (asr)

Dorsal part of the maxilla that excludes the maxillary body and contacts the nasal anteriorly and the lacrimal dorsally (Fig. 2A). Also known as the ascending process (e.g., [29], [36], [43]), posterodorsal process (e.g., [28], [44], [45]), nasal process (e.g., [27], [46], [47]), lacrimal process (e.g., [34]) and dorsal/ascending ramus of the nasal process [38].

#### Jugal ramus (jur)

Posterior part of the maxillary body situated below the antorbital fenestra (Fig. 2A). The jugal ramus, as used by several authors (e.g., [33], [34]), is also referred as the jugal process (e.g., [40], [41], [48]), posterior process (e.g., [31]), posterior ramus (e.g., [49]–[51]), subantorbital ramus (e.g., [29]), and subantorbital process (e.g., [38]).

#### Anteromedial process (amp)

Projection of bone on the medial surface of the maxillary body, on the anterodorsal corner of the anterior maxillary body, protruding anteriorly or anteroventrally to contact the premaxilla anteriorly, and the vomer and the opposite maxilla medially (Figs. 1C, 2C). The anteromedial process is also known as the rostromedial process (e.g., [25], [33]) and palatal process (e.g., [48], [52]–[54]).

### Walls, Shelves and Ridges

#### Lateral wall (law)

Bone surface laterally situated, covering the whole surface of the maxilla, from the ventral margin ventrally to the posterior tip of the ascending ramus dorsally, and bounding laterally the maxillary alveoli and different diverticula located within the maxilla (Fig. 2C). The lateral wall (lamina lateralis *sensu* [24]), as used by [36] and [41], is also known as the labial wall (e.g. [31], [55]) and lateral lamina (e.g., [31], [38], [41]).

#### Antorbital ridge (aor)

Low crest on the lateral surface of the maxilla, extending from the maxillary body to the ascending ramus, and bordering the lateral antorbital fossa anteriorly and ventrally (Fig. 1A).

#### Vestibular bulla (veb)

Convexity located on the anterodorsal margin of the maxillary body and the floor of the nasal vestibule, and corresponding to an inflated, thin-walled bony bubble of the anterodorsal portion of the promaxillary recess [24], [53] (Fig. 1A–C). The vestibular bulla (bulla vestibularis *sensu* [24]) can be perforated and opened to the external naris through a small foramen (the anterodorsal foramen). A vestibular bulla is noticeable in many non-avian theropods such as *Marshosaurus*, *Allosaurus*, *Sinraptor* [24], *Acrocanthosaurus* [49], *Proceratosaurus* [29], *Albertosaurus* [48], *Appalachiosaurus* ([56]:fig. 6A), *Byronosaurus* [57] and *Troodon* ([58]:fig. 2.1).

#### Medial wall (mew)

Bone surface medially situated, covering the surface of the maxilla dorsal to the nutrient groove (i.e., medial surface of the maxilla excluding the interdental plates), and bounding medially the different diverticula situated within the maxilla (Figs. 1C, 2C). The surface of the medial wall can be fenestrated at the level of the ascending ramus, and the maxillary antrum and promaxillary recess. Likewise, the medial wall ventral to the medial shelf can be undulated for receiving the dentary teeth if they are abutting against this surface when the jaws are closed (e.g., *Torvosaurus*, *Carcharodontosaurus*, *Tyrannosaurus*). The medial wall is also known as the medial lamina for some authors (e.g., [38], [50], [59]).

#### Medial shelf (mes)

Anterodorsally elongated ridge on the medial surface of the maxillary body, extending from the anteromedial process to the jugal ramus (and in some cases the jugal contact), and protruding medially to contact the opposite maxilla, palatine and, in some cases, vomer (Figs. 1C, 2C). Also known as the lingual bar (e.g., [41], [47]) or palatal shelf (e.g., [32], [34], [60]).

#### Lingual wall (liw)

Bone surface medially situated, covering the surface of the maxilla ventral to the nutrient groove and bounding each maxillary interdental plates medially, anteriorly and posteriorly (Figs. 1C, 2C). The lingual wall, as used by [31] and [36], is either formed by a row of separated interdental plates or a continuous interdental wall.

#### Interdental plate (idp)

Flat bony structure medial to the dental tooth row and attached to the lateral wall of the maxilla by a perpendicular and mediolaterally oriented lamina that separates each individual tooth socket (Fig. 2C). The interdental plates, also known as paradental plates [52], [61], [62], vary in size and morphologies and can either be separated by an interdental gap, or completely fused.

#### Interdental wall (idw)

Continuous medial wall ventral to the nutrient groove and formed by the fusion of interdental plates (Fig. 1C). The interdental wall is also known as the paradental lamina [29] or paradental shelf [63], and the array of unfused interdental plates present in many theropods does not constitute an interdental wall.

### Alveoli, Teeth and Margins

#### Maxillary alveoli (mal)

Tooth sockets located on the ventral margin of the maxilla (Fig. 3C2). They can be well-separated by the interdental plates, or merged to form an open groove like in troodontids.

#### Maxillary teeth (mx)

Teeth of the maxilla located within the alveoli (Figs. 1A–B, 2A). Due to the multiple generations of replacement teeth in the alveoli at one time, maxillary teeth, like those of the premaxilla and dentary, can be unerupted, semi-erupted and fully erupted.

#### Tooth root bulge (trb)

Crenulated margin of the anterodorsal rim of the jugal ramus resulting from the protrusion of the tooth roots into the antorbital fenestra (Fig. 3B1). A tooth root bulge (eminentia radices dentis *sensu* [24]) exists in some basal averostrans such as *Ceratosaurus* (USNM 4735; UMNH VP 5278; MWC 1.1) and *Marshosaurus* (UMNH VP 7824, 7825).

#### Alveolar margin (alm)

Ventral border of the maxilla along the maxillary tooth row (i.e., distance from the anterior point of the anteriormost maxillary alveolus to the posterior point of the posteriormost maxillary alveolus; Fig. 3D).

#### Ventral margin (vem)

Ventral border of the lateral wall of the maxilla, from the anteroventral corner of the anterior body, to the posteroventral extremity of the jugal ramus (Fig. 3D). The ventral margins of the lateral and medial walls do not always coincide, but the lateral margin extends more ventrally in the large majority of theropods (pers. obs.).

### Maxillary Contacts

#### Premaxillary contact (pmc)

Articular surface on the anterior margin of the maxillary body and receiving the premaxilla (Figs. 1B, 2C).

#### Jugal contact (juc)

Articular surface on the posterolateral or ventral surface of the jugal ramus of the maxilla and receiving the jugal bone (Figs. 1A, 2B).

#### Palatine contact (pac)

Articular surface along the medial shelf or the medial wall of the maxilla and receiving the palatine (Figs. 1C, 2C).

#### Nasal contact (nac)

Articular surface on the dorsal surface of the maxillary body and the anterior, dorsal, lateral and medial surface of the ascending ramus and receiving the nasal (Figs. 1C, E, 2C).

#### Lacrimal contact (lac)

Articular surface on the laterodorsal or dorsomedial surface of the ascending ramus and receiving the lacrimal (Figs. 1A, 2B).

### Fossae and Pneumatic Openings

#### Antorbital fossa (aofo)

Large depression surrounding and including the antorbital fenestra on the lateral and, in some cases, the medial surface of the maxilla. Its anterior, ventral and dorsal extensions are highly variable among theropods, covering most of the maxillary body in some basal tetanurans or reduced to a very short depression adjacent to the antorbital fenestra in some abelisaurids.

#### Lateral antorbital fossa (laof)

Depression surrounding the antorbital fenestra on the lateral surface of the maxilla (Figs. 1A, 2B). A peripheral rim and, in some case, a raised antorbital ridge along the lateral wall of the maxilla delimit the lateral antorbital fossa. The lateral antorbital fossa, corresponding to the external antorbital fenestra of [24], typically hosts the accessory antorbital fossae and fenestrae of the maxilla (e.g., promaxillary, maxillary, postmaxillary and pneumatic fenestrae and fossae) and pneumatic excavations. The lateral antorbital fossa is continuous with the antorbital fossa of the nasal, lacrimal and jugal in most of theropods.

#### Medial antorbital fossa (maof)

Depression surrounding the antorbital fenestra on the medial surface of the maxilla (Fig. 1C). The medial antorbital fossa is usually bordered by a peripheral step running from the maxillary body to the ascending ramus. It typically hosts some opening such as the posteromedial maxillary fenestra, several ventral pneumatopores and neurovascular openings. The medial antorbital fossa, which corresponds to the pneumatic fossa of [41], is continuous with the antorbital fossa of the palatine in most of theropods.

#### Maxillary fossa (mfo)

Depression variable in size and shape, homologous to the maxillary fenestra but bounded medially by a thick medial wall (Fig. 3B1–B2). The maxillary fossa, also known as the preantorbital fossa [43] and maxillary fenestra (e.g., [40], [41], [46], [62]), differs from the maxillary fenestra by being a shallow or deep and well-delimited depression that does not lead to a maxillary antrum. A maxillary fossa is present in coelophysoids (e.g., *Dracovenator*, *Zupaysaurus*, ‘*Syntarsus*’), *Ceratosaurus*, and non-spinosaurid megalosauroids (e.g., *Marshosaurus*, *Afrovenator*, *Dubreuillosaurus*, *Eustreptospondylus*, *Megalosaurus*, *Torvosaurus*). Given its size, shape and comparable location to this of coelophysoids and megalosauroids, the large depression located in the anterior corner of the lateral antorbital fossa is interpreted as the maxillary fossa in *Ceratosaurus*, *Limusaurus*, *Noasaurus*, *Masiakasaurus* and *Monolophosaurus*.

#### Promaxillary fossa (pmfo)

Depression variable in size and shape, homologous to the promaxillary fenestra but bounded medially by a thick medial wall. As for the maxillary fossa, the promaxillary fossa differs from the promaxillary fenestra in not leading to a promaxillary recess. A promaxillary fossa occurs in coelophysoids such as *Coelophysis*, *Dracovenator* and *Zupaysaurus*.

#### Pneumatic excavation (pne)

Fossa variable in size and shape but usually being a large ovoid or lanceolate depression located within the lateral or medial surface of the ascending ramus and bounded by the medial wall medially or lateral wall laterally (Figs. 1A, C, 2C, 3B1–B2). The pneumatic excavation (excavation pneumatica *sensu* [24]) can be fenestrated, as in *Eocarcharia* [50], and is generally located at mid-height of the ascending ramus, within the antorbital fossa. In some cases, it also communicates with other maxillary recesses situated more ventrally [24]. A pneumatic excavation exits in many theropods such as *Coelophysis* [42], *Ceratosaurus* (USNM 4735; MWC 1.1; UMNH VP 5278; Fig. 3B), *Sinosaurus* (KMV 8701), *Sinraptor* (IVPP 10600), *Yangchuanosaurus* (CV 00215, 00216), *Allosaurus* (UMNH VP 5393, 9168; USNM 8335), *Alioramus* (IGM 100-1844) and *Bambiraptor* (AMNH 30556).

#### Medial pneumatic complex (mpc)

Set of pneumatic excavations located within the anterior corner and dorsomedial surface of the medial antorbital fossa, and penetrating the ascending and jugal rami [41]. The medial pneumatic complex includes both anteromedial and posteromedial pneumatic recesses.

#### Anteromedial pneumatic recess (ampr)

Pneumatic excavation located within the anterior corner of the medial antorbital fossa and penetrating the ascending process of the maxilla (Fig. 3E–F). The anteromedial pneumatic recess, also known as the pneumatic excavation [40], [41], is homologous to the posteromedial maxillary fenestra but differs from the latter by not leading to a maxillary antrum. An anteromedial pneumatic recess can be observed in many megalosauroids such as *Piatnitzkysaurus* (PVL 4073), *Marshosaurus* (UMNH 7825), *Eustreptospondylus* (OUMNH J.13558), *Afrovenator* (MNN UBA1), *Megalosaurus* (OUMNH J.13506) and *Duriavenator* (BMNH R.332).

#### Ventromedial pneumatic recess (vmpr)

Pneumatic excavation located within the anteroventral corner or ventral part of the medial antorbital fossa, on the dorsomedial surface of the jugal ramus, and penetrating the jugal ramus of the maxilla (Fig. 3E–F; Fig. 3C2). The ventromedial pneumatic recess, also known as the pneumatic excavation [40], [41], is usually associated with an anteromedial pneumatic recess situated anterodorsally to it. A ventromedial pneumatic recess can be observed in several megalosauroids such as *Piatnitzkysaurus* (PVL 4073) and *Duriavenator* (BMNH R.332), and the tyrannosaurid *Tyrannosaurus* (CMNH 9380).

### Fenestrae

#### Antorbital fenestra (aofe)

Large opening posterior to the external naris and anterior to the orbital fenestra, and mostly delimited by the maxilla, jugal and lacrimal (Fig. 2B). Also known as the internal antorbital fenestra (fenestra antorbitalis interna *sensu* [24]), the external antorbital fenestra (fenestra antorbitalis externa *sensu* [24]) being delimited by the peripheral rim of the antorbital fossa [24].

#### Accessory antorbital fenestra (aafe)

Opening anterior to the antorbital fenestra within the anterior corner of the lateral antorbital fossa. Accessory antorbital fenestrae encompasses the promaxillary, maxillary, postmaxillary and pneumatic fenestrae. The accessory antorbital fenestra, also known as the accessory antorbital opening (e.g., [31]), is usually employed when it cannot be referred with certainty to the promaxillary or maxillary fenestra (e.g., [38], [50], [60], [64]). It also refers to the maxillary fenestra [65].

#### Maxillary fenestra (mfe)

Aperture variable in size and shape, but usually being a large, sub-circular opening, leading medially to the maxillary antrum or perforating the medial wall of the maxilla [24] (Figs. 1A, E, 2B, C, 3D). The maxillary fenestra [46], [66] (fenestra maxillaris *sensu* [24]), also known as the accessory foramen, second antorbital fenestra [67], second antiorbital fenestra [68], subsidiary antorbital fenestra [69], [70], and accessory antorbital fenestra (e.g., [65]), is situated within the anterior corner of the lateral antorbital fossa, at the base of the ascending ramus, posterior (and sometimes dorsal) to the promaxillary fenestra and anterior to the antorbital fenestra and the postmaxillary fenestra. Its presence has been noted in most nonavian neotetanurans (e.g., allosauroids, tyrannosauroids, compsognathids, ornithomimosaurs, therizinosauroids, oviraptorosaurs, deinonychosaurs), with perhaps the exception of *Erlikosaurus* [71].

#### Promaxillary fenestra (pmf)

Aperture variable in size and shape, but usually being a small slit-like opening, leading medially to the promaxillary recess, or in some cases, perforating the medial wall of the maxilla [24] (Figs. 1C, E, 2B, 3B1–C1). The promaxillary fenestra [72], (fenestra promaxillaris *sensu* [24]), also known as the promaxillary foramen (e.g., [29], [49], [59]), premaxillary fenestra (e.g. [30], [73], [74]) and tertiary antorbital fenestra (e.g., [38], [65]), is situated within the anterior corner of the lateral antorbital fossa, at the base of the ascending ramus and anterior to the maxillary fenestra. It is not always visible in lateral view, being concealed by the lateral wall of the maxilla and stuck up in the anterior corner of the lateral antorbital fossa. A slit-shaped promaxillary fenestra exists in many theropods such as *Herrerasaurus*, *Eodromaeus*, *Dilophosaurus*, Abelisauroidea, Megalosauroidea, Allosauroidea (e.g., *Allosaurus*, *Neovenator*), Tyrannosauroidea and most Maniraptoriformes, whereas a large discrete promaxillary fenestra can be observed in basal averostrans (e.g., *Ceratosaurus*, *Sinosaurus*), some allosauroids (e.g., *Sinraptor*, *Yangchuanosaurus*, *Acrocanthosaurus*, *Eocarcharia*), compsognathids (e.g., *Compsognathus*, *Scipionyx*) and possibly in oviraptorosaurs (e.g., *Incisivorosaurus*, *Citipati*, *Khaan*, see [75] for discussion on the accessory antorbital openings in Oviraptorosauria). Carcharodontosaurinae, some dromaeosaurids, and most derived Troodontidae seem to be devoid of a promaxillary fenestra ([38], pers. obs.), the maxillary and promaxillary fenestrae having most likely merged in Carcharodontosaurinae.

#### Pneumatic fenestra (pnf)

Aperture variable in size and shape, situated within the pneumatic excavation, and leading medially to a deep pneumatic recess within the ascending process, or in some cases, perforating the medial wall of the maxilla. The pneumatic fenestra, also known as the accessory fenestra [50], is present in the sinraptorid *Sinraptor* [24], [45], the basal carcharodontosaurids *Acrocanthosaurus* (right maxilla, [49]) and *Eocarcharia* [50], and the dromaeosaurid *Bambiraptor* (AMNH 30556).

#### Postmaxillary fenestra (ptmf)

Small sub-circular aperture situated within the antorbital fossa, between the maxillary fenestra and the antorbital fenestra (Fig. 3D). According to Larson [76], the postmaxillary fenestra, also known as the accessory maxillary fenestra [77] (small foramen along the ventral margin of the antorbital fossa of [78]), may result from depositional weathering or breakage. Its presence in many specimens of Tyrannosaurinae such as *Tyrannosaurus* (e.g., BHI 3033; LACM 23844; UCMP 118742), *Tarbosaurus* (ZPAL MgD-I/4) and *Zhuchengtyrannus* ([77]:fig. 2C–D) however makes this hypothesis unlikely. One or two small openings also exists within the antorbital fossa, between a large promaxillary fenestra (interpreted as such by [75]) and the antorbital fenestra, in the maxilla of the oviraptorid *Khaan* ([75], pers. obs.). Although the postmaxillary fenestra and these “postmaxillary” foramina occupy the same location within the antorbital fossa, they are not homologous.

#### Ventral maxillary fenestra (vmf)

Anteroposterioly elongated aperture situated on the antorbital body, beneath the lateral antorbital fossa. One or several ventral maxillary fenestrae have been noticed in several Oviraptoridae such as *Citipati* (IGM 100-978), *Khaan* (IGM 100-1127), *Conchoraptor* ([79]: fig. 8.1G) and an unpublished oviraptorid (MPC-D 100/4; [79]:fig. 8.1GE). These openings, referred to as the “additional accessory foramen” by [75], may not be pneumatic in nature, and may represent maxillary neurovascular foramina that are greatly enlarged, feeding the rhamphotheca and soft tissues of the jaw margin in oviraptorids (J. Headden pers. comm.). The ventral maxillary fenestrae may therefore be homologous to the row of maxillary circumfenestra foramina existing in other theropods. These large apertures do not seem to exist in any other nonavian theropod clade.

#### Posteromedial maxillary fenestra (pmmf)

Ventrodorsally elongated aperture delimited by the lateral wall of the maxilla laterally and the medial wall medially (Figs. 1C–D, 2C, 3A, C1–C2). The posteromedial maxillary fenestra, corresponding to the caudal fenestra of the maxillary antrum of [24] and used as such by several authors (e.g., [55]–[57]), is situated within the anterior corner of the medial antorbital fenestra and leads to the maxillary antrum. A posteromedial maxillary fenestra exists in spinosaurids (e.g., *Suchomimus*, *Spinosaurus*), allosauroids (*Sinraptor*, *Allosaurus*) and tyrannosauroids (e.g., *Alioramus*, *Tyrannosaurus*).

#### Dorsomedial maxillary fenestra (dmmf)

Elongated aperture located on the medial surface of the maxilla and perforating the dorsal wall of the maxillary antrum and, in some cases, promaxillary recess (Fig. 1F). The dorsomedial maxillary fenestra, corresponding to the subnarial fenestra of [46], is present in some Allosauroidea such as *Sinraptor* (IVPP 10600; [45]:fig. 4.12) and *Allosaurus* ([24], [46]; USNM 8335), the troodontid *Troodon* [58] and possibly some tyrannosaurids such as *Alioramus* [25].

#### Anteromedial maxillary fenestra (ammf)

Aperture within the anterior wall of the maxillary antrum (preantral strut) and leading to the promaxillary recess (Fig. 1E, 2C). An anteromedial maxillary fenestra, corresponding to the fenestra communicans *sensu* [24], exists in the majority of allosauroid and tyrannosauroid theropods.

#### Accessory maxillary fenestra (amf)

Aperture located within a fossa dorsomedial to the maxillary fenestra, dorsal to the posteromedial maxillary fenestra, and leading to the maxillary antrum (Fig. 3C2). Several accessory maxillary fenestrae have been noticed in one maxilla (CMNH 9380) of *Tyrannosaurus*.

#### Medial maxillary fenestra (mmf)

Subtriangular aperture perforating the medial wall of the maxilla and leading laterally to the maxillary antrum and promaxillary recess. The medial maxillary fenestra is delimited by the postantral strut posteriorly, the suprantral strut dorsally, the medial shelf ventrally and the anterior corner of the promaxillary recess anteriorly. Its presence has only been noticed in some basal allosauroids such as *Sinraptor* and *Allosaurus*.

### Antrum and Recesses

#### Maxillary antrum (man)

Large cavity located between the lateral and medial walls, anterior to the medial antorbital fossa, and communicating laterally with the maxillary fenestra [24] (Figs. 1C, E, 2C). The maxillary antrum [24] can also lead to the promaxillary recess via the anteromedial maxillary fenestra. The walls of the maxillary antrum can be reinforced by several struts (see below) that can be fenestrated. The maxillary antrum is also known as the maxillary sinus (e.g., [45], [46]) but the latter may refer to the sinus invading both maxillary antrum and promaxillary recess [24].

#### Promaxillary recess (pmr)

Cavity variable in volume within the medial wall, anterior to the maxillary antrum, and communicating laterally with the promaxillary fenestra (Figs. 1C, E, 2C). The promaxillary recess [24] is also known as the promaxillary sinus (e.g., [25], [37], [80]).

#### Epiantral recess (epi)

Small depression situated on the medial surface of the maxilla, posterodorsal to the maxillary fenestra, and excavating the anterodorsal surface of the interfenestral strut (Figs. 1C, E, 2C). An epiantral recess [24] is present in Allosauroidea (e.g., *Sinraptor*, *Allosaurus*) and Tyrannosauroidea (e.g., *Alioramus*, *Raptorex*, *Tyrannosaurus*, *Tarbosaurus*).

#### Interalveolar recess (iar)

Diverticula within the medial wall and the medial shelf and directed ventrally from the maxillary antrum and promaxillary recess, between the maxillary teeth (Fig. 3C1–2). An interalveolar recess, also known as the interalveolar pneumatic recess (recessus pneumatici interalveolares *sensu* [24]) is only present in Tyrannosauridae like *Alioramus*, *Albertosaurus* and *Tyrannosaurus* ([24], [25], pers. obs.).

### Foramina and Grooves

#### Subnarial foramen (snf)

Small opening variable in outline and located between the premaxilla and maxilla, below the external naris (Fig. 1A). The subnarial foramen corresponds to the maxilla-premaxillary fenestra of [67], [68] and the subnarial fenestra of [81].

#### Anterodorsal foramen (adf)

Small opening located on the anterodorsal margin of the maxilla and perforating the dorsomedial wall of the promaxillary recess. The anterodorsal foramen is present in some troodontids such as *Troodon* [58].

#### Nutrient groove (nug)

Furrow running anterodorsally on the medial surface of the maxillary body and hosting the nutrient foramina (Figs. 1C, 2C). The nutrient groove, also known as the groove for the dental lamina (e.g., [35], [37], [50]) and the paradental groove (e.g., [29], [36], [82]), corresponds to the junction between the interdental plates and the medial wall. Due to the fact that the medial wall slightly overlap the interdental plates medially, the nutrient groove is delimited by the interdental plates laterally and the medial wall medially, and by both interdental plates and medial wall dorsally and ventrally. A similar groove, the paradental groove, exists on the medial surface of the dentary, ventral to the interdental plates.

#### Nutrient foramina (nuf)

Small openings on the interdental plates, at the level of the nutrient groove, permitting the unerupted teeth to be innervated by blood vessels inside their alveoli [49] (Figs. 1C, 2C). Also known as nutrient notches (e.g., [47], [83]), suprainterdental plate foramina [27], or dental foramina (e.g., [53], [68], [84]).

#### Interdental gap (idg)

Ventrodorsally elongated groove separating each interdental plate while they are unfused (Fig. 2C).

#### Maxillary neurovascular foramina (mnf)

Small openings located on the lateral surface of the maxillary body and permitting the passage of blood vessels to innervate the lips and cheeks.

#### Maxillary alveolar foramina (maf)

Row of neurovascular foramina parallel with and adjacent to the ventral margin of the maxilla (Figs. 1A, 2B).

#### Maxillary median foramina (mmf)

Neurovascular foramina randomly distributed and located in between the rows of maxillary alveolar and circumfenestra foramina (Fig. 1A).

#### Maxillary circumfenestra foramina (mcf)

Row of neurovascular foramina parallel with and adjacent to the ventral rim of the antorbital fossa (Figs. 1A, 2B).

### Maxillary Struts

#### Promaxillary strut (prms)

Lamina or column separating the promaxillary fenestra from the maxillary fenestra (Fig. 2B). The promaxillary strut (pila promaxillaris *sensu* [24]), as called by several authors (e.g., [32], [49], [59]), is also known as the promaxillary pila (e.g., [85], [86]).

#### Interfenestral strut (ifs)

Bone wall separating the maxillary fenestra from the antorbital fenestra (Figs. 1A, 2B, C). The interfenestral strut (pila interfenestralis *sensu* [24]), is also known as the interfenestral bar (e.g., [56], [59], [63], [83], [85]).

#### Postmaxillary strut (ptms)

Bone surface separating the maxillary fenestra from the postmaxillary fenestra (Fig. 3D). Only present in Tyrannosauridae (e.g., BHI 3033, LACM 23844, ZPAL MgD-I/4).

#### Postantral strut (poas)

Pillar of bone delimiting the posteromedial maxillary fenestra medially, and the maxillary antrum posteromedially (Figs. 1C–F, 2C, 3A, C2). The postantral strut (pila postantralis *sensu* [24]) can be fenestrated by the posteromedial maxillary fenestra, allowing communication of the antorbital cavity and the maxillary antrum [24].

#### Suprantral strut (suas)

Ridge reinforcing the dorsal wall of the maxillary antrum dorsomedially (Fig. 1E). The suprantral strut can be perforated by the dorsomedial maxillary fenestra [24].

#### Preantral strut (pras)

Pillar of bone separating the maxillary antrum from the promaxillary recess (Figs. 1C, E, 2C). The preantral strut, corresponding to the maxillary septum *sensu* [46], can be doubled (i.e., presence of lateral and medial preantral struts) when the promaxillary fenestra is internal (i.e., within the maxilla and the maxillary antrum) as in *Allosaurus* (Fig. 1E).

## Results

### Systematic Paleontology

Dinosauria Owen, 1842 [87]

Saurischia Seeley, 1887 [88]

Theropoda Marsh, 1881 [89]

Tetanurae Gauthier, 1986 [66]

Megalosauroidea Fitzinger, 1843 [90]

Megalosauridae Fitzinger, 1843 [90]

*Torvosaurus* Galton & Jensen, 1979 [91]

#### Revised diagnosis

Megalosauroid theropod with very shallow maxillary fossa (i.e., maxillary fossa forming a poorly delimited concavity in the anterior corner of the lateral antorbital fossa) [61], protuberant ridge below the maxillary fossa, in the ventral part of the anterior corner of the lateral antorbital fossa, interdental wall making up one-half the medial surface of the maxillary body (modified from [27]), expanded fossae in posterior dorsal and anterior caudal centra forming enlarged and deep pneumatic openings [61], highly ossified puboischiadic plate [61], and distal expansion of ischium with prominent lateral midline crest and oval outline in lateral view [61].

*Torvosaurus tanneri* Galton & Jensen, 1979 [91]

Galton & Jensen ([91]:figs. 1, 2, 3A, G, L, 4A–F, 4I–N; 6–7, 8H); Jensen ([92]:figs. 1–4A–D, E–F, 5A–F, H); Britt ([27]:figs. 2–24)

1988 *Megalosaurus tanneri*; [91]; [93], p. 282.

1992 *Edmarka rex* gen. nov.; [94]:figs. 1, 3, 7, 10, 12–15.

1997 ‘*Brontoraptor*’ sp. gen. nov.; [95]:figs. 1–9, 10A–E, 11A–E, 12–13A, 14–15A, 16A–H, 17 (*nomen nudum*).

#### Lectotype

BYU-VP 2002, left humerus ([27]).

#### Paralectotype

BYU-VP 2002, the rest of left and right forelimbs ([27]).

#### Referred material

(from [61]) BYU-VP 2003, 2004, 2005, 2006, 2007, 2008, 2016, 2017, 4838, 4853, 4860, 4882, 4883, 4884, 4890, 4908, 4951, 4952, 4976, 4998, 5004, 5005, 5008, 5009, 5010, 5020, 5029, 5077, 5086, 5092, 5110, 5129, 5136, 5147, 5242, 5254, 5276, 5277, 5278, 5279, 5280, 5281, 5286, 8907, 8910, 8937, 8938, 8966, 8982, 9013, 9090, 9108, 9120, 9121, 9135, 9136, 9141, 9142, 9143, 9144, 9152, 9161, 9162, 9163, 9249, 9620, 9621, 9622, cranial and postcranial elements [27]; TATE 401, 1002–1005 (*Edmarka rex*), jugal, scapulocoracoid, and ribs [94]; TATE 0012, with 0012-11 formally 1003, (‘*Brontoraptor*’), atlas, axis, sacrum, caudal vertebrae, chevrons, scapula, coracoids, ilium, pubis, ischium, femur, tibia, fibula [95]; FMNH PR 3060, three midline fragments of gastralia, right metacarpal III, right manual phalanx III-2, left metatarsals II–IV, left pedal phalanx I-1 [96].

#### Locality and horizon

Dry Mesa Quarry, Montrose County, Calico Gulch Quarry, Uncompahgre Plateau, Moffit County, and Meyer site, Garden Park, north of Cañon City, Fremont County, Colorado; Carnegie Quarry, Dinosaur National Monument, Uintah County, Utah; Gilmore Quarry N and Quarry 6, Freezeout Hills, Carbon County, and Nail and Louise Quarries, Como Bluff, Albany County, Wyoming, USA; Salt Wash and Brushy Basin Members, Morrison Formation; Kimmeridgian-Tithonian, Late Jurassic [61], [96].

#### Diagnosis

Megalosauroid theropod with a protuberant ridge on the anterior part of the medial shelf, posterior to the anteromedial process, and an interdental wall falling short relative to the lateral wall (i.e., ventral margin of the interdental wall much more dorsal than the ventral margin of the lateral wall) and formed by the fusion of interdental plates with broad V-shaped ventral margin.

**Figure 4:**
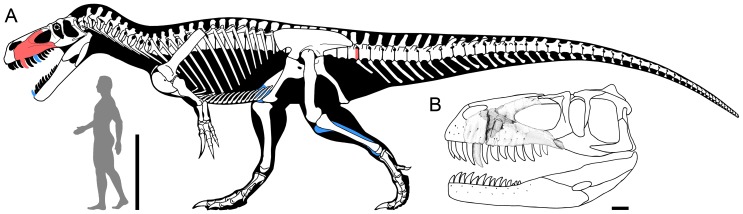
Reconstruction of *Torvosaurus gurneyi* in lateral view. A, Skeletal reconstruction of *Torvosaurus gurneyi* in lateral view illustrating, in red, the elements present in the holotype specimen (ML 1100) and, in blue, the elements tentatively assigned to this species (artwork by Scott Hartman, used with permission and modified; drawing of man by Carol Abraczinskas, University of Chicago, used with permission). B, Skull reconstruction of *Torvosaurus gurneyi* in lateral view illustrating the incomplete left maxilla(ML 1100) of the holotype specimen (artwork by Simão Mateus, used with permission and modified). Scale bars = 1 m (A) and 10 cm (B).

*Torvosaurus gurneyi* Hendrickx & Mateus 2014 sp. nov. urn:lsid:zoobank.org:act:189C1060-7887-4837-9E30-870079E2B2B9 (Fig. 4).

*Torvosaurus tanneri* Mateus et al. ([4]:fig. 6).

#### Holotype

**Figure 5:**
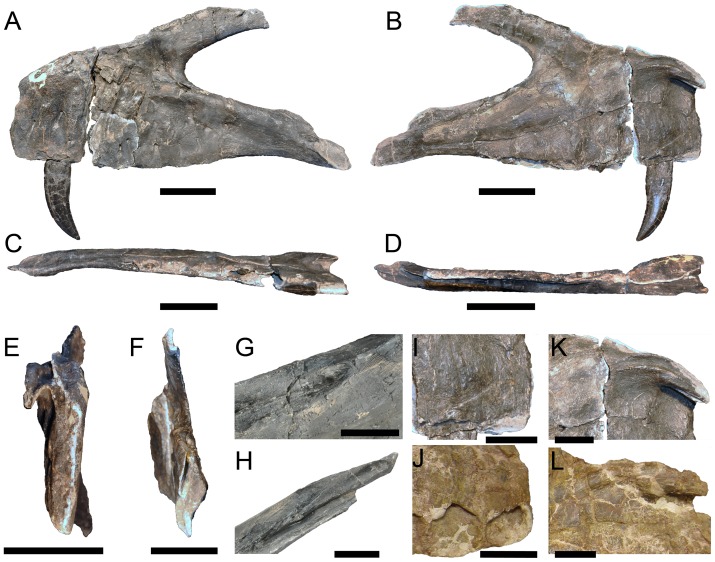
Maxilla of *Torvosaurus gurneyi* (ML 1100) and comparison with *T. tanneri*. Incomplete left maxilla of the holotype specimen of *Torvosaurus gurneyi* (ML 1100) in **A**, lateral; **B**, medial; **C**, ventral; **D**, dorsal; **E**, anterior; **F**, posterior views with details of **G**, Anterodorsal margin of jugal ramus in dorsomedial view; and **H**, Posterior part of jugal ramus in dorsal view. **I–J**, Anterior part of interdental wall of **I**, *T. gurneyi*; and **J**, *T. tanneri* (BYUVP 9122) in medial view. **K–L**, Anteromedial process of **K**, *T. gurneyi*; and **L**, *T. tanneri* (BYUVP 9122) in medial views. Scale bars = 10 cm (A–H), 5 cm (G–L).

**Figure 6:**
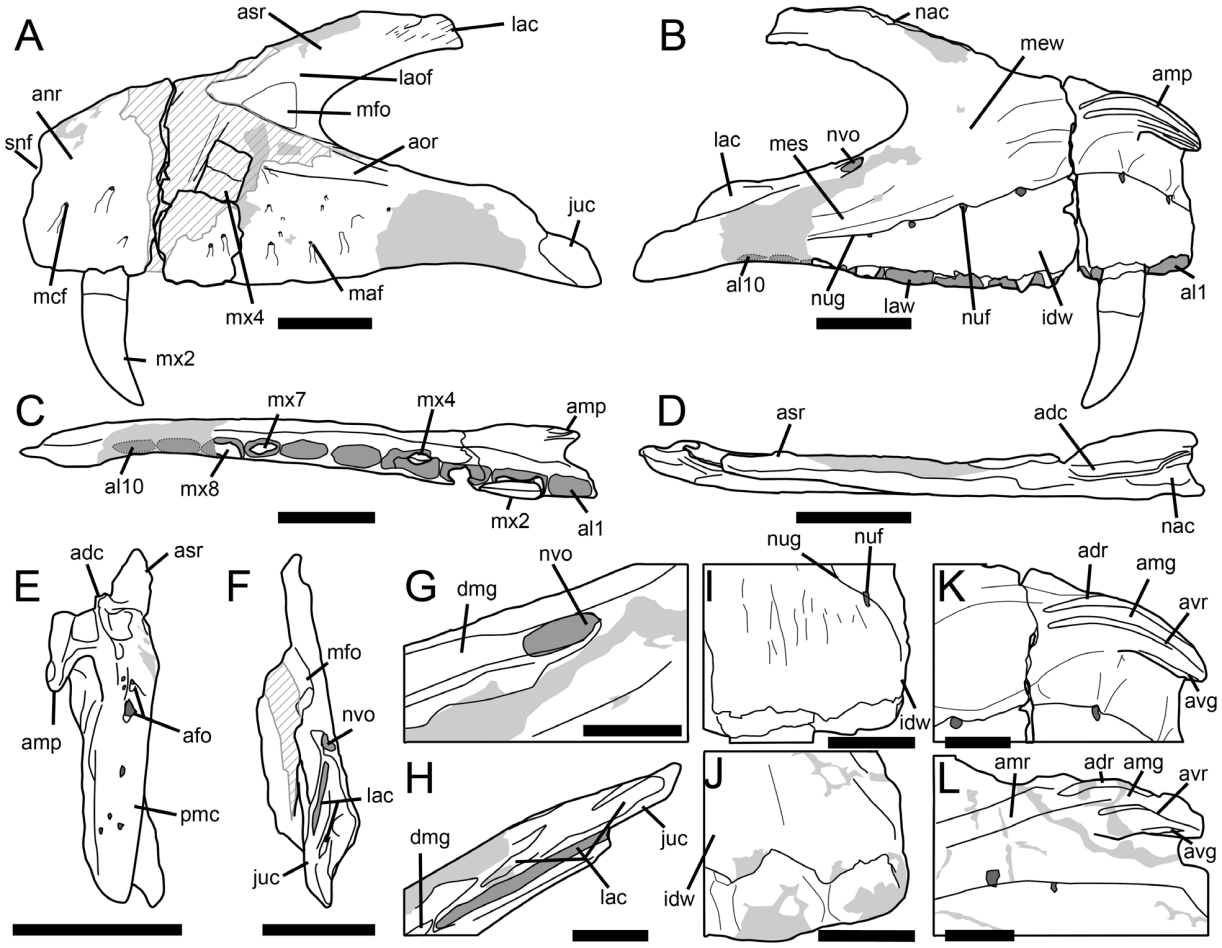
Maxilla of *Torvosaurus gurneyi* (ML 1100) and comparison with *T. tanneri*. Interpretive line drawing of the left maxilla of the holotype specimen of *Torvosaurus gurneyi* (ML 1100) in **A**, lateral; **B**, medial; **C**, ventral; **D**, dorsal; **E**, anterior; **F**, posterior views with details of **G**, anterodorsal margin of jugal ramus in dorsomedial view; and **H**, posterior part of jugal ramus in dorsal view. **I–J**, Interpretive line drawing of the anterior part of interdental wall of **I**, *T. gurneyi*; and **J**, *T. tanneri* (BYUVP 9122) in medial view. **K–L**, Interpretive line drawing of the anteromedial process of **K**, *T. gurneyi*; and **L**, *T. tanneri* (BYUVP 9122) in medial views. Hatched areas represents missing parts, light grey tone indicates reconstructed part, and dark grey tone corresponds to the pneumatopores, foramina, and alveoli, with alveoli 9 and 10 being reconstructed. Abbreviations: **adc**, anterodorsal crest; **adr**, anterodorsal ridge of the anteromedial process; **afo**, anterior foramina; **al**, alveolus; **amg**, anteromedial groove of the anteromedial process; **amp**, anteromedial process; **amr**, anteromedial ridge; **anr**, anterior ramus; **aor**, antorbital ridge; **asr**, ascending ramus; **avg**, anteroventral groove of the anteromedial process; **avr**, anteroventral ridge on the anteromedial process; **dmg**, dorsomedial groove; **idw**, interdental wall; **juc**, jugal contact; **lac**, lacrimal contact; **laof**, lateral antorbital fossa; **law**, lateral wall; **maf**, maxillary alveolar foramina; **mcf**, maxillary circumfenestra foramina; **mes**, medial shelf; **mew**, medial wall; **mfo**, maxillary fossa; **mx**, maxillary teeth; **nac**, nasal contact; **nuf**, nutrient foramina; **nug**, nutrient groove; **nvo**, neurovascular opening; **pmc**, premaxillary contact; **snf**, subnarial foramen. Scale bars = 10 cm (A–H), 5 cm (G–L).

**Figure 7:**
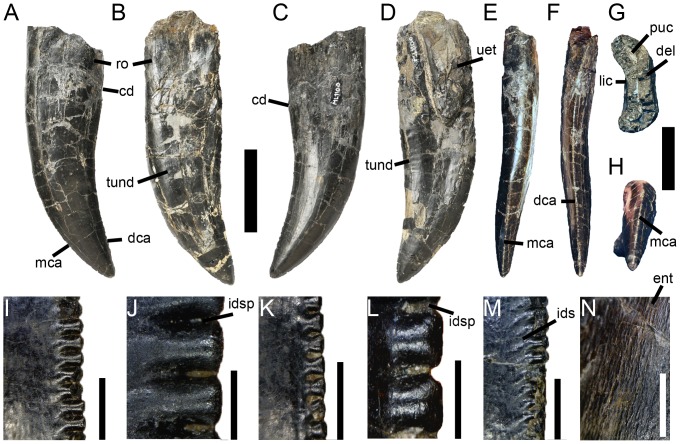
Dentition of *Torvosaurus gurneyi* (ML 1100). **A, C, E–H**, Second maxillary tooth; and **B, D**, third non-erupted maxillary tooth of the holotype specimen of *Torvosaurus gurneyi* in **A–B**, labial; **C–D**, lingual; **E**, mesial; **F**, distal; **G**, basal; and **H**, apical views. **I–J**, Distal; and **K–M**, mesial denticles of the second maxillary tooth in lateral view. **M**, Distal serrations showing the interdenticular sulci; and **N**, enamel texture of the third non-erupted tooth in labial view. Abbreviations: **cd**, cervix dentis; **dca**, distal carina; **del**, dentine layer; **ent**, enamel texture; **ids**, interdenticular sulci; **idsp**, interdenticular space; **mca**, mesial carina; **lic**, lingual concavity for the erupting tooth; **puc**, pulp cavity; **ro**, root; **uet**, unerupted tooth; **und**, transversal undulation. Scale bars = 5 cm (A–F), 3 cm (G–H), 3 mm (I, K, M–N), 1 mm (J, L).

**Figure 8:**
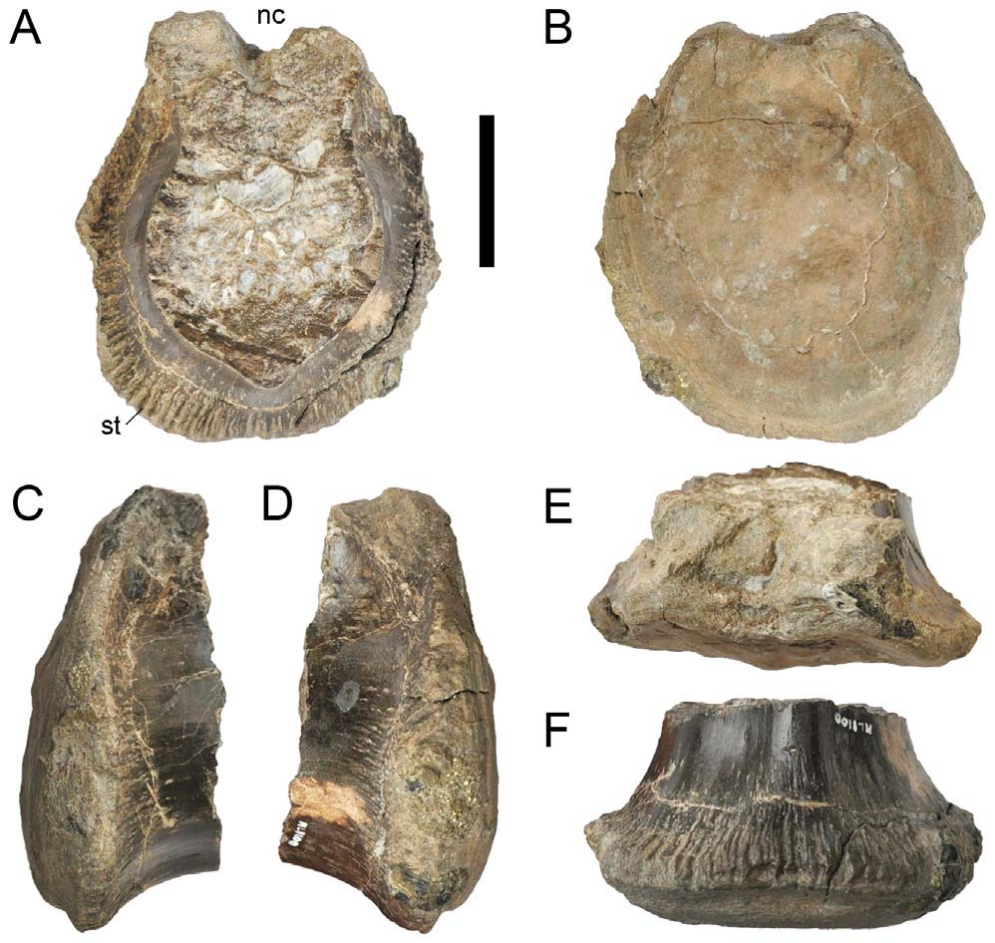
Caudal vertebra of *Torvosaurus gurneyi* (ML 1100). **A–D**, Posterior part of an anterior caudal centrum of the holotype specimen of *Torvosaurus gurneyi* (ML 1100) in **A**, anterior; **B**, posterior; **C**, right lateral; **D**, left lateral; **E**, dorsal; and **F**, ventral views. Abbreviations: **nc**, neural canal; **st**, striation. Scale bar = 5 cm.

ML 1100, an incomplete left maxilla (Figs. 4B, 5–6) bearing one erupted tooth and one unerupted tooth (Fig. 7), and the posterior portion of a proximal caudal vertebra (Fig. 8).

#### Referred material

**Figure 9:**
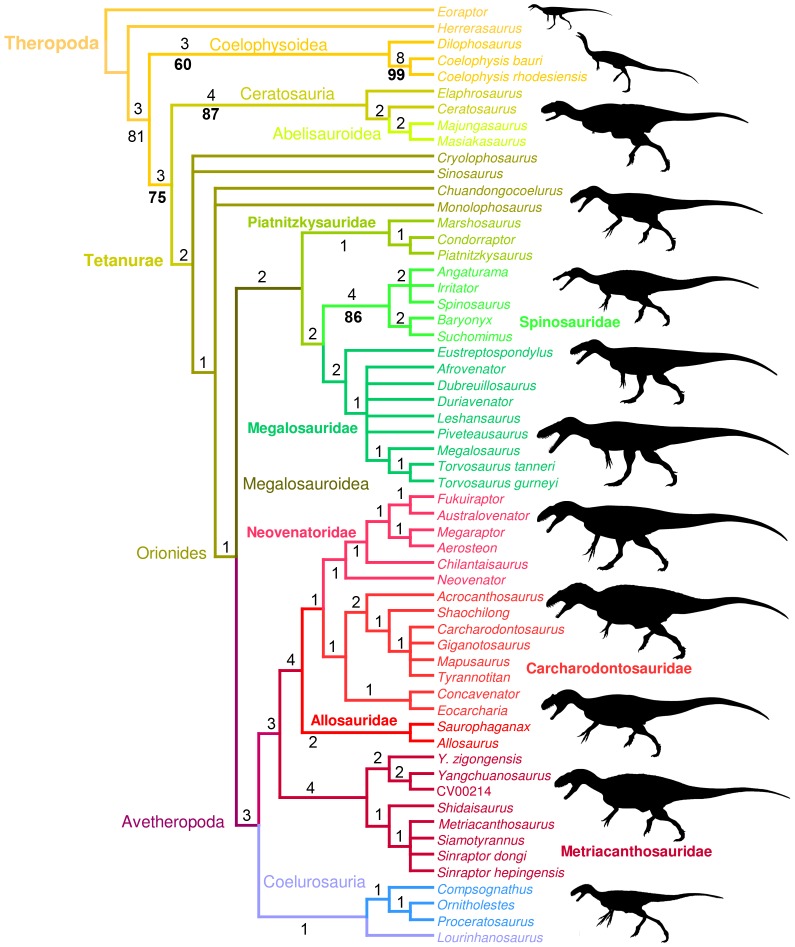
Cladogram of basal Theropoda and phylogenetic position of *Torvosaurus gurneyi*. Strict consensus cladogram from 71 most parsimonious trees after pruning *Magnosaurus*, *Poekilopleuron*, *Streptospondylus* and *Xuanhanosaurus* from the full set of most parsimonious trees. Initial analysis used New Technology Search using TNT v.1.1 of a data matrix comprising 353 characters for two outgroup (*Eoraptor* and *Herrerasaurus*) and 60 nonavian theropod taxa. Tree length = 1022 steps; CI = 0.414, RI = 0.685. Bremer support values are in regular and bootstrap values are in bold. Dinosaur silhouettes by Scott Hartman (all but Metriacanthosauridae; used with permission) and Gregory S. Paul (Metriacanthosauridae; used with permission).

ALT-SHN.116, a portion of a right maxilla [8]. ML 962, a mesialmost shed tooth ([6]:fig. 9), FUB PB Ther 1, a lateral tooth, ML 430, an incomplete tibia [7], ML 632, a partial femur [4], and ML 1186, cranial and postcranial material of embryos [9], are tentatively referred to *T. gurneyi*.

#### Type

Cliffs of Praia da Vermelha, Lourinhã, Portugal. Porto Novo-Amoreira Member, Lourinhã Formation, Upper Kimmeridgian, Upper Jurassic [21].

#### Etymology

In honor of the paleoartist James Gurney, creator of the utopic world of *Dinotopia*.

#### Diagnosis

Megalosauroid theropod with maxillae bearing fewer than eleven teeth and possessing fused interdental plates with straight ventral margin forming an interdental wall nearly coincidental with the lateral wall of the maxillary body. Differs from *Torvosaurus tanneri* by fewer than eleven maxillary alveoli, the absence of interdental plates terminating ventrally by broad V-shaped points and falling short relative to the lateral wall, the absence of a protuberant ridge on the anterior part of the medial shelf, posterior to the anteromedial process, and the coincidental posterior extension of the dorsal and medial ridges of the anteromedial process.

#### Taphonomy

The specimen was found in beach eroded boulders that fell from the sea cliff. The bones did not show any signs of articulation, except the maxilla preserving the teeth in situ. The elements are not visibly compressed or deformed. The caudal centrum, directly associated with the maxilla and showing some *Torvosaurus* characters, has three patched of pyrite encrustations and attached to charcoal. This suggests taphonomical or depositional anoxic conditions.

### Description

#### Maxilla

A fairly complete and undistorted left maxilla (Fig. 5) was collected in Praia da Vermelha in June 2003 [4]. Some bone surfaces on the lateroposterior side of the anterior ramus and on the anterodorsal corner of the lateral antorbital fossa are missing. Likewise, some bone fragments on the medial surface of the jugal ramus, including the posteriormost alveoli, are absent. The maxilla is also broken in two pieces at the level of the third alveolus, and a fragment of the lateral surface of the maxilla can be removed at the level of alveolus 4, allowing examination of a complete unerupted tooth (Figs. 5–6A). Only a fully-erupted tooth, the second maxillary tooth, is preserved, and the crown tips of the third and six alveoli are visible. The maxilla is thick and massive, with a short posterodorsally angled ascending ramus and a high anteroposteriorly elongated maxillary body (Fig. 5–6A; Table 1). The ventral margin of the maxillary body is weakly sigmoid, with a convex, almost straight, ventral margin of the anterior body, and a concave ventral margin of the jugal ramus.

**Table 1:** Measurements of left maxilla of the holotype of *Torvosaurus gurneyi* (ML 1100).

	Measurements (mm)
Anteroposterior length of maxilla:	612
Dorsoventral depth of maxilla at the posteriormost point of the ascending ramus:	274
Dorsoventral depth of maxillary body at the level of the step delimiting the anterior ramus and ascending ramus:	226
Anteroposterior length of antorbital body:	310
Anteroposterior length of jugal ramus:	299
Dorsoventral depth of jugal ramus at the anterior margin of antorbital fenestra:	170
Dorsoventral depth of ascending ramus along its main axis:	237
Dorsoventral depth of anterior margin of maxillary body:	122
Anteroposterior length of anteromedial process	115
Anteroposterior length of jugal contact:	83
Dorsoventral depth of interdental wall at the level of the third alveolus:	106
Basoapical length of second maxillary tooth, root included:	138
Basoapical length of third non-erupted maxillary tooth, root included:	165

The anterior body of the maxilla is longer than the jugal ramus (Table 1), yet the posterior extremity of the jugal ramus is broken and the posterior part may have extended further posteriorly. Nevertheless, the anterior body is high and about one third higher than the jugal ramus at its anteriormost part (Figs. 5–6A). The dorsal rim of the anterior body is convex and anteroventrally inclined. It mostly includes an anterior ramus which is demarcated by concave step on the anterodorsal margin of the maxilla. Both anterior ramus and preantorbital body have similar anteroposterior extensions along the maxillary body. The anterior ramus is particularly high and elongated, and its posterior rim is concave whereas its ventral margin is straight. The anterior rim of the anterior ramus is high (about two thirds of the anterior ramus height in its highest part), subvertical, and perpendicular to the ventral margin of the maxillary body. The outline of the anterior margin is irregular and roughly sigmoid in lateral view, the ventral half is convex while the dorsal half is concave due to the presence of a ventrodorsally wide subnarial foramen. The dorsal margin of the anterior ramus bears a thin crest, the anterodorsal crest (Figs. 5–6D–E), running from alveoli 1 to 3 and adjacent to the anteromedial process. This narrow crest is slightly medially inclined and taller in its anterior part. It also shows an undulating dorsal rim. The anteromedial process and the anterodorsal crest both delimit a deep anteroposteriorly extended groove that received the ventral articular surface of the nasal. The nasal contact of the anterior ramus is narrow and shallow in its posterior part, anterior to the ascending ramus, and gets wider and deeper at the level of the anteromedial process.

The premaxillary contact is located on the anterior rim of the anterior ramus. It is a rather simple articulation that corresponds to a roughly flat but uneven surface. The premaxillary articulation bears two large foramina on its dorsalmost part, the smaller one being situated dorsolateral to the larger one, in the dorsolateral corner of the premaxillary contact. These two anterior foramina (Fig. 5–6E) lead to the subnarial foramen, an aperture that is posteriorly delimited by the maxillary body and the maxillary contact of the premaxilla anteriorly. The subnarial foramen is not clearly visible but corresponds to a wide concavity on the anterolateral margin of the maxillary body, at the dorsalmost third of the premaxilla contact. Additional foramina are visible medial to the anterior foramina, and along the ventral half of the premaxillary contact. These additional foramina are minute in size, and smaller than the two anterior foramina (Figs. 5–6E). Two pits also exist on the dorsalmost part of the premaxillary contact, between the lateral wall of the anterior ramus and the medial wall attached to the anteromedial process. These two pits accommodated the bifurcated maxillary process of the premaxilla. In anterior view, the medial margin of the premaxillary articulation is straight whereas the lateral margin is convex. In medial view, the lateral wall of the anterior ramus extends slightly further anteriorly than the medial wall.

The jugal ramus is sub-triangular in outline and tapers gently ventroposteriorly. The surface of the jugal ramus bears a small and shallow concavity on its anterolateral margin, at the level of alveolus 6. This concavity is bounded ventrally by the antorbital ridge. A wide furrow is visible on the dorsomedial surface of the jugal ramus, ventral to the antorbital fenestra. This groove most likely corresponds to a neurovascular opening serving for the passage of the maxillary branch of the trigeminal nerve (O. Rauhut, pers. comm.). The neurovascular opening (Fig. 6B–G) runs from the lacrimal contact of the maxilla to the level of the eighth alveolus, just below the antorbital fenestra. The groove is shallow anterior to the lacrimal contact but penetrates deeply inside the medial wall of the jugal ramus in its anterior part. The jugal articulates with the posterior extremity of the jugal ramus, along a smooth articular surface on the lateroventral margin of the jugal ramus. The anterior rim of the jugal contact is parabolic in outline, and the main axis of the articulation is inclined ventroposteriorly. Its ventral rim corresponds to a narrow groove penetrating the lateral wall of the jugal ramus.

A second articulating surface, the lacrimal contact, appears on the posteromedial margin of the jugal ramus, posterior to the neurovascular opening, and at two thirds of the jugal ramus (Fig. 6B). The lacrimal contact extends along the posterior extremity of the jugal ramus, posterior to the eighth alveolus. The lacrimal contact covers around one half of the jugal ramus. The dorsal rim of the lacrimal contact forms a convexity on the dorsal margin of the jugal ramus, and the ventral part consists of a very deep slit inside the jugal ramus, so that the maxillary contact of the jugal corresponded to very thin articular structure (Fig. 6F–H). The lacrimal contact also includes a second furrow running along the dorsomedial rim of the jugal ramus, medial to the deep split and dorsal to the lateral part of the lacrimal articulation(Fig. 6H). The latter is bounded laterally by the lateral wall of the maxillary body on its anterior part, its posterior part being adjacent to the jugal contact on the lateroposterior surface of the jugal ramus. The main axis of the lacrimal contact is directed posteroventrally, parallel to the ventral rim of the antorbital fenestra. Similar to the jugal contact, the lacrimal contact of the jugal ramus is a simple suture i.e., it is not reinforced by a series of grooves and rugosities.

The ascending ramus forms a wing-like structure diverging from the maxillary body to an angle of around 30° with the ventral margin (Figs. 5–6A). The ascending ramus is short compared to the anteroposterior extension of the maxillary body (Table 1), but its posterior extremity is broken and must also have extended further posteriorly (Fig. 4). Although some parts of the anterior margin of the ascending ramus are missing, the anterior and posterior rims are sub-parallel along the anterior part of the ramus but the anterodorsal rim abruptly changes orientation at two thirds of the process so that the jugal ramus tapers posteriorly. The medial surface of the ascending ramus is slightly concave, and a small depression appears on the posteromedial surface of the ascending ramus, on the centre of the process. Unlike other articular surfaces on the maxilla, the lacrimal contact of the ascending ramus is not clearly delimited. A few parallel ridges are visible on the lateroposterior surface of the ascending ramus, and the lacrimal contact is bounded by a sharp ridge parallel to the rim of the antorbital fenestra on its ventromedial surface. A furrow is also present on the posterolateral margin of the ascending ramus and was bordering the anterior rim of the lacrimal. This wide groove runs diagonally on the posterior extremity of the ascending ramus and is bounded by a short crest anteriorly. Two shallow concavities appear anterior to this ridge and their main axis is sub-parallel to the diagonal furrow.

The anteromedial process of the maxilla is complete, protuberant and clearly-visible on the anterodorsal corner of the anterior body, immediately ventral to its dorsal rim, and to a certain distance dorsal to the nutrient groove (Figs. 5–6B, K). This process sweeps gradually and tapers ventrally at the level of the first alveolus. It bears two large and parallel ridges separated by a wide groove on its medial surface, and a shallow and straight groove on its ventromedial surface (Fig. 6K). Both ventral and dorsal ridges get flared at the level of the third alveolus posteriorly, and the wide groove they delimit gets deeper anteriorly. The anteromedial process does not extend further than the third alveolus posteriorly, and only expands slightly further than the anterior rim of the maxillary body anteriorly.

The medial shelf is poorly delimited. It corresponds to a wide but shallow ridge running on the medial wall of the maxillary body, from the anteromedial process to the posterior part of the jugal ramus (Figs. 5–6B). The medial shelf is clearly sigmoid i.e., it is convex along the jugal ramus and concave along the anterior ramus. A subtle flattened surface is visible at the level of the fourth alveolus, posteroventral to the anteromedial process. There is no trace of articulating surface for the palatine on the preserved medial shelf. The palatine may have been in contact with the medial margin of the maxillary body posterior to the eighth alveolus, yet the palatine articulation may have just been eroded more anteriorly.

The surface of the medial wall is smooth all along the maxilla. It bears two concavities just ventral to the anteromedial process, at the level of the first and second alveoli (Fig. 6K). The anterior concavity is significantly wider than the posterior one and subcircular in outline. The posterior depression is weakly ventrodorsally elongated and subrectangular in outline. These two deep pits accommodated two large crowns of the dentary while the jaws of the animal were closed. A deep depression occurs on the anterodorsal surface of the anterior body, beneath the anterior part of the anteromedial process. This depression is bounded dorsally by a thin convex lamina linking the anteromedial process to the anterior ramus. The medial wall is neither fenestrated nor perforated at the base of the ascending ramus, and there is no trace of medial antorbital fossa and medial pneumatic complex.

The nutrient groove is distinct and forms a strong step between the medial wall and the interdental plates (Figs. 5–6B). The groove is sigmoid and subparallel to the medial wall, and strongly curves ventrally at the level of the second alveolus. It bears seven clearly-visible nutrient foramina at the level of each alveolus, exactly aligned with their centre (Figs. 5–6B). The nutrient foramen of the third, eighth and more posterior alveoli are not preserved. These dental foramina increase in size with the fourth alveoli and then decrease in dimension more posteriorly. They are lanceolate to elliptical in outline, the largest one being almost subcircular at the level of the fourth alveolus. The nutrient foramina weakly penetrates the medial wall dorsally.

The interdental plates are completely fused to form a continuous lamina along the medial surface of the maxillary body (Figs. 5–6B, I). Their height increases along the two first alveoli, then their ventrodorsal extension decreases posterior to alveolus 3. They are particularly high at the level of the second and third alveolus, being twice higher than wide, and the ventral extend of the interdental wall is as far ventral as the lateral wall of the maxillary body. The medial surface of the interdental plates is irregular and rugose, and the presence of faint grooves running ventrodorsally on the ventral margin can be noticed (Fig. 6I).

The antorbital fenestra is almost perfectly parabolic in outline i.e., the curvatures of the ventral and dorsal rims of the antorbital fenestra are subsymmetrical, the ventral margin being only slightly wider ventrally. The medial antorbital fossa is absent but the lateral antorbital fossa extends far anterior on the maxilla. The extension of the lateral antorbital fossa is important on the ascending ramus but limited to the dorsalmost part of the maxillary body. The lateral antorbital fossa is bounded ventrally by a wide and poorly delimited antorbital ridge on the dorsal part of the jugal ramus (Figs. 5–6A). The antorbital ridge is missing in the dorsal part of the anterior body and all along the ascending ramus so that it is not possible to know the exact extension of the antorbital fossa in its anteriormost corner.

No promaxillary or maxillary fenestrae are present within the lateral antorbital fossa. Nevertheless, a subtriangular depression is visible on the anterior corner of the antorbital fossa, just anterior to the anteriormost point of the antorbital fenestra and dorsal to the antorbital ridge of the anterior body. Due to its large size, shape and location, the subtriangular depression is here interpreted as homologous to the maxillary fossa (or imperforated maxillary ‘fenestra’ of [41]). A single accessory antorbital fossa occupying most of the anterior corner of the lateral antorbital fossa has usually been interpreted as being a maxillary fossa/fenestra rather than a promaxillary fossa/fenestra, and the latter is only large when associated to the maxillary fenestra (pers. obs.). It is very likely that the antorbital ridge was forming a lateral rim on the anteroventral part of the ascending ramus, delimiting a deep recess within the anterior corner of the lateral antorbital fossa. The posteriormost part of a poorly defined ridge is visible dorsal to the antorbital ridge, on the anterodorsal part of the jugal ramus, at the level of the fourth alveolus. Although this ridge is strongly damaged more anteriorly, its posterior rim can be followed from the antorbital fenestra to the anteriormost part of the maxillary recess.

The texture of the lateral surface of the maxilla is not rugose or sculptured, but the lateral surface of the maxillary body is pierced by a series of large, deep and well-delimited neurovascular foramina. A wide groove, parabolic in outline in some cases, extends ventrally from each neurovascular foramina which penetrate the lateral wall of the maxilla dorsally. Although many neurovascular foramina are missing due to damage of the lateral bone surface, two rows of neurovascular foramina are clearly visible and both run anteroposteriorly on the maxillary body, parallel to the ventral margin. The ventral row, which includes the maxillary alveolar foramina, is adjacent and slightly dorsal to the ventral margin of the maxillary body, whereas the dorsal row, that encompasses the circumfenestra foramina, is centrally positioned on the maxillary body and runs shortly dorsally to the row of alveolar foramina.

Eight maxillary alveoli are distinctly visible along the maxillary body, and the preserved posterior part of the jugal ramus does not preserve any alveolus (Figs. 5–6C). The tooth row extends anterior to the jugal contact, and the largest tooth-sockets are located at mid-length of the maxillary body, the largest alveolus being the sixth one. The alveoli are well-separated and elliptical in outline all along the tooth row.

#### Dentition

The second fully erupted maxillary tooth and the third unerupted tooth (Figs. 5–7) are well preserved and allow the crown and denticles morphology to be investigated comprehensively. The second erupted tooth is complete and undistorted while the unerupted one has been crushed inside its alveolus and the labial and lingual surfaces are damaged. The apical part of a third unerupted tooth appears on the basolingual surface of the unerupted tooth, inside the fourth alveolus (Fig. 7D). This second unerupted crown correspond to the third generation of teeth in the maxilla.

The crowns are ziphodont (i.e., blade shaped, labiolingually compressed, distally curved and having serrated carinae), large (crown height >100 mm; Table 2) and strongly elongated (crown height ratio >2.5; [6]). They are significantly recurved distally and bear prominent carinae mesially and distally. In distal view, the crown and the distal carina of the erupted tooth are gently sigmoid in outline, with the root curving lingually from the crown (Fig. 7F). The basolabial surface of the erupted crown is mesiodistally concave and this depression allows the accommodation of an unerupted crown lingually. The distal carina extends to the cervix whereas the mesial carina does not reach the root and gets flared at one third of the crown (Fig. 7E). Both carinae are centrally positioned on the crown although the basal part of the mesial carina tends to get slightly offset at mid-height of the crown. The cross section outline of the crown is reniform at the cervix, lanceolate at one third of the crown and elliptical more apically. The external surface is particularly well-preserved and shows a clear braided and basoapically oriented texture of the enamel (Fig. 7N). Although not present on the erupted crown, subtle transversal undulations (“enamel wrinckles” *sensu* [97]) are observable on the basal half of the unerupted crown, on both labial and lingual sides (Fig. 7B, D). The undulations are more pronounced adjacent to the distal carina on the lingual surface of the crown. Only the basal part of the root of the second maxillary tooth is preserved. The root clearly shows a deep concavity on its lingual surface for receiving the unerupted crown. Such lingual concavity is also present on the other teeth of the maxilla as the cross section outline in the root of these teeth is clearly reniform.

**Table 2:** Measurements of maxillary teeth of the holotype of *Torvosaurus gurneyi* (ML 1100).

	Measurements (mm)
**Second erupted maxillary tooth**	
Crown base length (CBL)	45.52
Crown base width (CBW)	16.4
Crown height (CH)	106.4
Apical length (AL)	118.57
Mid-crown length (MCL)	33.1
Mid-crown width (MCW)	16.8
Extension of mesial denticles from cervix (MDE)	55.51
**Third unerupted maxillary tooth**	
Crown base length (CBL)	45.65
Crown base width (CBW)	?
Crown height (CH)	116.98
Apical length (AL)	128.59
Mid-crown length (MCL)	39.54
Mid-crown width (MCW)	?
Extension of mesial denticles from cervix (MDE)	46.38

The denticles are large and coarse, with an average of eight denticles per five millimetres on both carinae (Fig. 7I–M; Table 3). The crown apex is damaged in the erupted crown, but the serrations are clearly crossing the apex of the unerupted tooth. In the second maxillary crown, there is a density of ten to eleven denticles per five millimetres basodistally, eight denticles at mid-crown and six to seven serrations per five millimetres apically for both carinae, so that the denticle size increases from the base to the apex (Table 3). Mesial and distal denticles of both erupted and unerupted crown differ in their morphology and elongation. The distal denticles are chisel-like in shape (i.e., denticles with a sharp edge) in mesial and distal views and finger-like in shape (i.e., horizontal subrectangular denticles with convex labial and lingual surfaces) in lateral view (Fig. 7I–J). They extend perpendicularly from the distal margin of the crown and possess narrow but deep interdenticular space. The external margin of each denticle is symmetrically to asymmetrically convex but never hooked apically. Pronounced and clearly-visible interdenticular sulci are present all along the distal carina (Fig. 7M). These grooves curve basally from each interdenticular space and are particularly long at mid-crown. They are shorter more basally and apically, being very short to absent near to the cervix and the apex. Unlike the distal serrations, the mesial denticles have subquadrangular to vertical subrectangular profile in lateral view (Fig. 7K–L). They are either perpendicular to or weakly apically inclined from the mesial margin of the crown, and their external margin is symmetrically to asymmetrically convex. The interdenticular space is deep and tends to be basoapically wider at mid-height and narrower at the level of the apex in some denticles, creating an elliptical to lanceolate outline of the interdenticular space. The interdenticular sulci are short or totally absent from mesial serrations. On the unerupted tooth where they are clearly visible, they are short to absent on the lingual side but totally absent on the labial surface of the crown.

**Table 3:** Number of denticles in maxillary teeth of the holotype of *Torvosaurus gurneyi* (ML 1100).

	Denticles (per 5 mm)
**Second erupted maxillary tooth**	
Mesioapical denticles (MA)	6
Mesial denticles at mid-height (MC)	8
Mesiobasal denticles (MB)	/
Distoapical denticles (DA)	7
Denticles at mid-height (DC)	8
Distobasal denticles (DB)	11
**Third unerupted maxillary tooth**	
Mesioapical denticles (MA)	6
Mesial denticles at mid-height (MC)	7
Mesiobasal denticles (MB)	/
Distoapical denticles (DA)	6
Denticles at mid-height (DC)	8
Distobasal denticles (DB)	10

Several isolated bone fragments, including the proximal portion of a rib, a strongly damaged fragment of a long bone and a caudal vertebra, have been uncovered from the same area of the maxilla. Nevertheless, only the caudal vertebra comes from the same spot and was directly associated with the maxilla. Likewise, its size, preservation and taxonomic identification allows assigning the caudal vertebra to the same specimen with confidence.

#### Caudal vertebra

The posterior third of a caudal centrum (Fig. 8) with about 57 mm is preserved. We interpret this bone as a proximal caudal vertebra based on comparisons with the *T. tanneri* holotype (BYU-VP 13745), in particular based on the lack of an elongated pneumatic foramen extending along most of the centrum length, shallow chevron facets and the flattened to sub-convex articular surface. The general outline of the posterior view forms a large ellipse about 131 mm tall and 120 mm wide (Table 4). The articular facet is moderately flat; however, in the middle of the surface there is a tuberosity projecting posteriorly, and shallow depressions below and above it are also visible (Fig. 8E). The lateral and ventral margin of the centrum have well-defined striations that run anteroposteriorly on the centrum, being deeper and pronounced in the ventral half the centrum (Fig. 8A, F). These sulci are up to 20 mm long, 2 mm wide, and 1.2 mm deep, but the dimensions vary. These dimensions provide a density of 3.5 ridges per centimetre. The ventroposterior corner of the centrum is expended but with no clear individual facet for the chevrons. The posterior rim if the centrum possesses circular striations. There is a horizontal transversal groove on the posteroventral corner of the centrum between the ventralmost rim of the centrum and the platform of the articular facet. This gives a salient aspect to the posterior region of the centrum, but this can also be interpreted as a sub-convexity of this facet. The anterior broken transversal section has an amphora-like outline. This outline is produced ventrally by a rounded ridge-like midline crest, and dorsally by the posterodorsal corner of the centrum that is slightly narrower transversely, giving a constriction of the amphora-like outline (Fig. 8A). The bone is compact towards the periost, and camellate in the anterior part of the centrum. The neural canal is narrow ventrally which gives a V-shape at the cross-section in anterior view but broader and U-shaped in posterior view (Fig. 8A–B). The pedicel width is equivalent to the neural canal at mid-level of the neural canal, where it is broken dorsally. The pedicels reach the posteriormost facet of the centrum. The general surface of the bone is lustrous in the lateral and ventral surface of the centrum, but matt on the posterior facet. If complete, the centrum would be moderately excavated, giving a hourglass outline in ventral view.

**Table 4:** Measurements of proximal caudal vertebra of the holotype of *Torvosaurus gurneyi* (ML 1100).

	Measurements (mm)
Dorsoventral height of centrum at the level of the neural canal:	129
Dorsoventral height of centrum at its maximum height:	145
Transverse width of centrum:	121
Anteroposterior length of centrum:	52

### Phylogenetic Analysis

ML 1100 was previously assigned to *Torvosaurus tanneri* by Mateus et al. [4] based on an antorbital tooth row, the absence of a maxillary fenestra (antorbital foramen of [4]) and pneumatisation on the ascending ramus, and the posterior orientation of the ascending ramus of the maxilla. In order to confirm the phylogenetic affinities of this specimen, a cladistic analysis was performed using the datamatrix of Carrano et al. [61]), the most recent and exhaustive analysis focusing on relationships of basal Tetanurae. The datamatrix includes 60 ingroup taxa and two outgroups (*Eoraptor* and *Herrerasaurus*) coded in 353 unordered and equally weighted characters [61]. Following personal observation of the maxilla in basal tetanurans, one character was modified from Carrano et al. [61] and two additional characters were created (see Text S1). A total of 36 characters were coded for the maxilla, two for the interdental plates, nine for the dentition and one for the caudal vertebra. TNT v1.1 [98] was employed to search for most-parsimonious trees (MPTs). As a first step, the matrix was analysed under the ‘New Technology search’ with the ‘driven search’ option, TreeDrift, Tree Fusing, Ratchet, and Sectorial Searches selected with default parameters, and stabilizing the consensus twice with a factor of 75. The generated trees were then analysed under traditional TBR (tree bisection and reconnection) branch [98]. Bremer support [99] and Reduced Cladistic Consensus Support Trees [100] were calculated with TNT by saving 10,000 suboptimal trees up to 10 steps longer than the MPTs. The consistency and retention indexes as well as the Bremer and relative Bremer supports were calculated using the “stats” and the “aquickie” commands, respectively.

The cladistic analysis yielded 93 MPTs, 1033 length, with a consistency index of 0.404 and a retention index of 0.677 for the strict consensus tree. The tree mirrors to a large degree the topology obtained by Carrano et al. [61]) and retrieved ML 1100 and *Torvosaurus tanneri* as sister taxa. The clade of Megalosauria [61] was however badly resolved and a reduced consensus approach [100]–[102] was used by excluding a posteriori four wildcard taxa with a lot of missing data (*Magnosaurus*, *Poekilopleuron*, *Streptospondylus* and *Xuanhanosaurus*). The topology of the resulting consensus tree is similar to the consensus tree obtained when excluding a priori the four taxa (Fig. 9), and the tree displays a few polytomies, mostly in the clade of Megalosauridae and Carcharodontosaurinae. Nevertheless, all major clades of Tetanurae were found resolved and the *Torvosaurus* taxa are still closely related, forming the sister clade of the taxon *Megalosaurus* (Fig. 9). Following the result of the cladistic analysis, ML 1100 can confidently be assigned to the taxon *Torvosaurus*. The maxilla ML 1100 indeed belongs to a theropod based on the combination of a subnarial foramen and very large ziphodont teeth bearing coarse denticles, a tetanuran due to its anteroposteriorly long anterior ramus, the presence of a maxillary recess (i.e., either a maxillary fenestra or a maxillary fossa) within the lateral antorbital fossa, and a tooth row extending anterior to the orbit. In also pertain to a megalosaurid by the presence of a maxillary fossa, to the clade encompassing *Megalosaurus* and *Torvosaurus* by the tall interdental plates (ventrodorsal depth relative to the anteroposterior width >1.8 [61]), and to the *Torvosaurus* by the shallow maxillary fossa, limited ventral extension of the lateral antorbital fossa on the maxillary body, and fused interdental plates forming an interdental wall [61].

## Discussion

**Figure 10:**
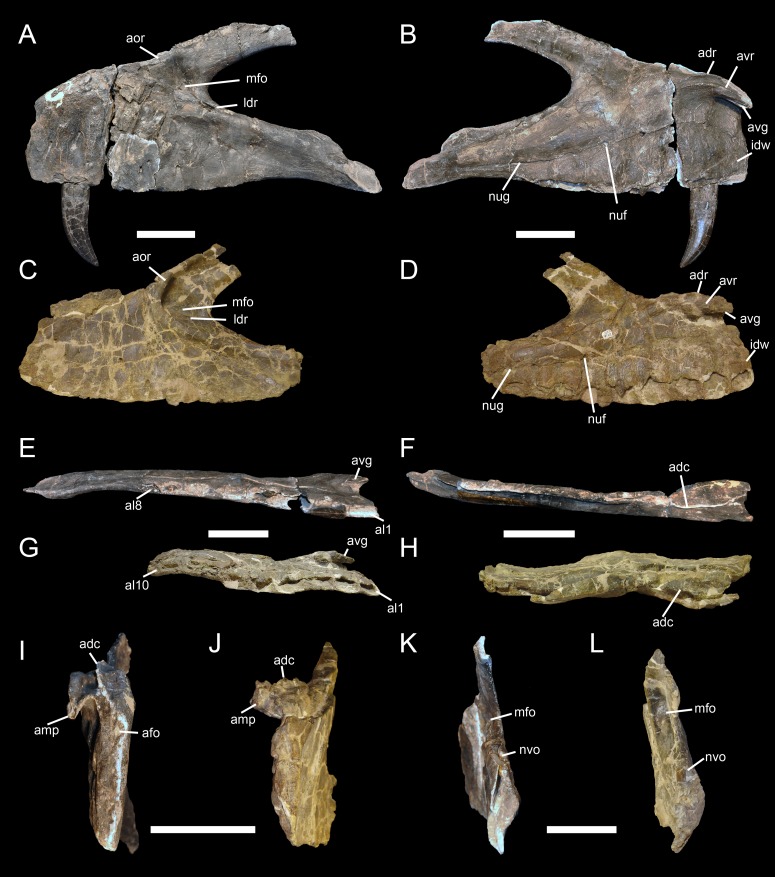
Comparison of the maxillae of *Torvosaurus gurneyi* and *Torvosaurus tanneri*. Left maxillae of the holotype specimen of *Torvosaurus gurneyi* (ML 1100) in **A**, lateral; **B**, medial; **E**, ventral; **F**, dorsal; **I**, anterior; and **K**, posterior views. Left maxillae of a specimen referred to *Torvosaurus tanneri* (BYUVP 9122) in **C**, lateral; **D**, medial; **G**, ventral; **H**, dorsal; **J**, anterior; and **L**, posterior views. Abbreviations: **adc**, anterodorsal crest; **adr**, anterodorsal ridge of the anteromedial process; **afo**, anterior foramina; **al1**, first alveolus; **al8**, eighth alveolus; **al10**, tenth alveolus; **amp**, anteromedial process; **aor**, antorbital ridge; **avg**, anteroventral groove of the anteromedial process; **avr**, anteroventral ridge on the anteromedial process; **idw**, interdental wall; **ldr**, laterodorsal ridge within the anterior corner of the lateral antorbital fossa; **mfo**, maxillary fossa; **nuf**, nutrient foramina; **nug**, nutrient groove; **nvo**, neurovascular opening. Scale bars = 5 cm.

The maxillae of ML 1100 and the referred specimen of *T. tanneri* BYU-VP 9122 share striking similarities (Fig. 10). Not only their anatomy is very close but they also share similar size and angles of rami. Many other features are common between ML 1100 and BYU-VP 9122, namely, large and elongated teeth with coarse denticles (8 denticles per 5 mm or less), a shallow subtriangular maxillary fossa at the base of the ascending ramus, an ascending ramus angled at 30° from the ventral margin, an anteroposteriorly oriented ridge ventral to the shallow maxillary fossa within the lateral antorbital fossa, and very tall fused interdental plates that are perforated by large nutrient foramina at the level of the nutrient groove. Therefore, the Portuguese specimen clearly belongs to the taxon *Torvosaurus* first described from the Kimmeridgian-Tithonian of North America [91]. The two taxa also share similar stratigraphical range as the Portuguese specimen is late Kimmeridgian in age, and its American counterpart has been recorded in late Kimmeridgian to late Tithonian deposits [61]. Nevertheless, they were geographically separated by thousands of kilometres and the proto Atlantic epicontinental sea was restraining the European *Torvosaurus* to the Iberian Meseta [21]. If the assignation of ML 1100 to *Torvosaurus* is hardly doubtable, it is legitimate to assess its affiliation to the species *T. tanneri* given the paleogeographical context.

A detailed comparison of ML 1100 with BYU-VP 9122 (and ML 1186, a cast of BYU-VP 9122 deposited at the Museu of Lourinhã) allows highlighting some differences between the two maxillae (Figs. 6–7I–L, 10). One of the most notable was observed by Mateus et al. [4] and concerns the maxillary tooth count. Eleven alveoli have been noticed by Britt [27] for BYU-VP 9122 and, according to this author, there were up to 12 or 13 maxillary teeth based on the intersection of the medial wall and ventral margin. On the other hand, the Portuguese specimen possesses eight maxillary alveoli, with a maximum number of ten teeth [4]. Although maxillary alveoli gradually decrease in size in theropods, the spacing between them remains the same (pers. obs.). In megalosauroids, the last alveoli never occupy less than 50% of the size of the largest alveoli (pers. obs.), so that the presence of more than two alveoli in the missing section of the jugal ramus is very unlikely, and there were almost certainly no more than ten teeth in ML 1100. Examination of ML 1186 does not clearly reveal the presence of an eleventh alveoli, and only ten alveoli, with nine complete and the posteriormost one incomplete, could only be observed. Based on the posterior intersection of the boundary between the interdental wall and the ventral margin, we evaluate the total number of alveoli to eleven or twelve in *T. tanneri*. This therefore corresponds to a slightly closer tooth count of BYU-VP 9122 from ML 1100. Although tooth count is commonly used for taxonomic purpose by many authors in nonavian theropods (e.g. [6], [39], [41], [61], [62]), variation in the number of maxillary alveoli occurs through ontogeny (e.g., [13], [48]), between individuals of the same species (e.g., [46], [53], [103], [104]), and even between left and right maxillae of a same specimen (e.g., [32], [104], [105]). Tooth count should therefore be cautiously employed for synapomorphic purpose. Nevertheless, it is interesting to highlight that ML 1100 is the only megalosauroid possessing fewer than eleven teeth on the maxilla and, with the exception of the toothless ceratosaur *Limusaurus* [106] and the primitive theropod *Daemonosaurus* [107], the only non-coelurosaurian theropod with such a feature (pers. obs.). The maxilla of *Noasaurus*, interpreted as having 10 to 11 maxillary teeth by [108], in fact possesses 12 to 13 alveoli (pers. obs.).

Another difference between the American and European specimens is the ventral extension of the interdental plates relative to the lateral wall as well as the morphology of the ventral terminations of the interdental plates (Fig. 5–6I–J). In ML 1100, the interdental plates extend almost as far ventral as the lateral wall, whereas the interdental plates of *T. tanneri* fall short and end well dorsal of the lateral wall of the maxillary body. This later feature is considered to be a synapomorphical character of the clade encompassing *Torvosaurus* and *Megalosaurus* by Benson [41] and Carrano et al. [61]. It can also be observed in other theropods such as the tyrannosauroids *Guanlong*, *Daspletosaurus* and *Tyrannosaurus* and the allosauroids *Allosaurus* and *Neovenator* (see Table S1; pers. obs.). Britt [27] remarked that this character may be due to crushing but examination of ML 1186 seems to reveal that the interdental plates genuinely end well dorsal to the lateral wall of the maxilla. Nonetheless, it is difficult to know whether this feature can variate ontogenetically, intraspecifically or can genuinely distinguish two taxa. Based on very large and similar size of their maxillae, ML 1100 and BYU-VP 9122 clearly belong to animals of the same size and ontogenetic stage, and most likely fully adult individuals of more than nine meters (see below), so that ontogenetic variation cannot be taken into consideration. The maxillae of the different specimens of *Dilophosaurus wetherilli* (UCMP 37303, TMM 43646-1; Fig. S1), *Ceratosaurus nasicornis* (UMNH VP 5278; MWC 1), *Majungasaurus crenatissimus* (FMNH PR 2100, 2278), *Marshosaurus bicentesimus* (UMNH VP 7824, 7825; CMNH 21704), *Megalosaurus bucklandii* (BMNH R.8303; OUMNH J13506, 13559), *Allosaurus fragilis* (AMNH 600, 851; BYU-VP 2008; UMNH VP 5393, 9168, USNM 8335) and *Tyrannosaurus rex* (CMNH 9380; FMNH PR 2081; BHI 3033) all show similar ventral extension of the interdental plates. On the other hand, the two species of *Carcharodontosaurus*, *C. saharicus* (SGM Din–1) and *C. iguidensis* (MNN IGU2) can be differentiated on this aspect as the interdental plates of the former extend more ventral than the latter (pers. obs.). Based on this observation, the ventral extension of the interdental plate relative to the lateral wall may genuinely variate interspecifically and this feature is therefore considered to be a synapomorphical character differentiating the two species of *Torvosaurus*. To our knowledge, the presence of an interdental wall coincidental with the lateral wall of the maxillary body is an autapomorphical feature of *T. gurneyi* among Megalosauroidea.

As noted by Britt ([27]:17), the interdental plates of the maxilla also “terminate ventrally in broad, V-shaped points” in BYU-VP 9122 (Fig. 5J). On the contrary, the ventral rim of the interdental plates are straight and continuous all along the interdental wall in ML 1100 (Fig. 5I). A V-shaped margin of the interdental plates is common among theropods and can be observed in the noasaurid *Masiakasaurus*, the abelisaurids *Rugops* and *Indosuchus*, the megalosauroids *Marshosaurus*, *Piatnitzkysaurus*, *Eustreptospondylus*, *Duriavenator* and *Afrovenator*, the allosauroids *Allosaurus*, *Neovenator*, *Sinraptor* and *Mapusaurus*, and the tyrannosaurids *Alioramus*, *Tarbosaurus* and *Tyrannosaurus* (see Table S1). Subrectangular interdental plates can however be seen in the ceratosaurs *Ceratosaurus*, *Noasaurus*, *Aucasaurus* and *Majungasaurus*, the megalosaurid *Megalosaurus*, the allosauroid *Shaochilong*, and the tyrannosauroid *Eotyrannus* (pers. obs.; Table S1). Variation in the ventral margin of the interdental plates does not seem to occur among mature individuals of the same species, with perhaps the exception of *Mapusaurus* in which the V-shaped of the interdental plates seems to be much more pronounced in MCF-PVPH-108.169 than in MCF-PVPH-108.115 ([34]:fig. 2B–D). However, anterior interdental plates are badly preserved in MCF-PVPH-108.115 and the posterior ones show the distinct V-shaped condition (pers. obs.). A similar variation also exists in the two species of *Carcharodontosaurus* (pers. obs.). In *C. iguidensis*, the ventral margin of the fused interdental plates are clearly V-shaped whereas in *C. saharicus*, although many of them are not intact, the plates tend to have a much straighter ventral margin. Surprisingly, in *Dilophosaurus wetherilli* the morphology of the interdental plates differ significantly between the youngest juvenile TMM 43646-1, the adult specimen UCMP 77270 and the immature individuals UCMP 37302 (holotype) and 37303 (paratype; [42]:fig. 36; see File S1). In TMM 43646-1 and UCMP 77270, the plates are separated, subquadrangular to vertical subrectangular and the ventral margin is clearly V-shaped whereas the type specimens possess fused interdental plates that are horizontally rectangular with a straight ventral margin (Fig. S1). Whether fusion and variation in the interdental plates morphology may occur throughout ontogeny, such intraspecific variability of the interdental plates seems very unlikely and we therefore estimates that TMM 43646-1 and UCMP 77270 may represent a different taxon of *Dilophosaurus wetherilli*, as it was already suggested ([83]; see File S1). We therefore consider the straight ventral margin of the interdental plates as a potential synapomorphical character of the clade encompassing *Megalosaurus*+*Torvosaurus* (under ACCTRAN optimization), and interdental plates with V-shaped ventral margin are therefore the plesiomorphic condition in tetanurans and megalosauroids.

The morphology of the medial wall and the anteromedial process also differ between the American and European *Torvosaurus* (Figs. 5–6K–L). BYU-VP 9122 displays a protruding ridge corresponding to the anterior part of the medial shelf (Fig. 5L). It extends from the posterodorsal part of the anteromedial process and gets flared to the level of alveolus 4. This ridge is absent from ML 1100 where only a low and wide anteroposteriorly oriented convexity corresponding to the anterior part of the medial shelf is observable (Fig. 5K). The medial shelf of the jugal ramus is more prominent in *T. tanneri* than in ML 1100 but the latter displays a low crest centrally positioned on the medial shelf, a feature absent in BYU-VP 9122. The ventral ridge of the anteromedial process also extends more anterior than the dorsal one, and only to the level of the second alveolus in BYU-VP 9122. On the other hand, the two main ridges of the anteromedial process of ML 1100 get flared at the same level posteriorly. In Megalosauroidea, this condition is shared with *Marshosaurus*, *Piatnitzkysaurus*, *Eustreptospondylus*, *Afrovenator* and *Megalosaurus* whereas in *Duriavenator*, *Dubreuillosaurus* the dorsal ridge of the anteromedial process extends further posteriorly than the ventral one (pers. obs.). Likewise, the groove delimited by the two ridges of the anteromedial process is notably wider in BYU-VP 9122 than in ML 1100. Furthermore, the posterior nutrient foramina are conspicuously larger in BYU-VP 9122 and, in this specimen, the anterior rim of the maxillary body is more inclined posteriorly, the anterior part of the ventral margin smoothly curves dorsally, and the parabolic outline of the antorbital fenestra is ventrodorsally wider. Again, it is difficult to know whether these differences between the American and European specimens exist inter- or intraspecifically, but some of them can certainly be considered as intraspecific variations.

As noted, some anatomical differences can be observed between ML 1100 and BYU-VP 9122, mostly in the morphology of the medial shelf and interdental plates. The presence of fused interdental plates forming a wall coinciding with the lateral wall of the maxilla is an autapomorphical character of ML 1100 among megalosauroids and, to our knowledge, this feature does not vary intraspecifically. Likewise, the protruding ridge posterior to the anteromedial process of BYU-VP 9122 seems to be an autapomorphy of *Torvosaurus tanneri* among non-coelurosaurs theropods, and the absence of this feature in ML 1100 supports its affiliation to a different taxon. Finally, the geographical context of the European specimen of *Torvosaurus*, which seems to have been isolated on the Iberian Meseta in the Kimmeridgian [21], favours this option, and we therefore refer the Portuguese specimen to a new species of *Torvosaurus*, *Torvosaurus gurneyi*.

*Torvosaurus gurneyi* provides additional information on the maxilla anatomy of *Torvosaurus*. The dorsal margin of the ascending ramus is smoothly convex and the anterodorsal rim of the ascending ramus makes a step at two thirds of the process so that the ventral part of the ascending ramus tapers posteriorly. There is a small convexity on the dorsal margin of the jugal ramus, at two thirds of it, where the lacrimal articulates with the maxilla medially. Likewise, the lacrimal articulation of the maxilla corresponds to a deep slit within the jugal ramus. The presence of a neurovascular opening penetrating the maxilla on the dorsomedial margin of the jugal ramus, at the level of the eighth alveolus, can also be noted and represents an autapomorphy for the taxon *Torvosaurus*. This opening is also present in BYU-VP 9122 but not well visible due to crushing so that Britt [27] did not mention it.

### Size and Paleogeographical Implications

With a minimum length of 612 mm, the maxilla of *Torvosaurus gurneyi* pertains to a very large individual positioned at the apex of the food chain in the Late Jurassic ecosystem of Iberia. The maxilla occupies 52% (*Allosaurus*) to 61% (*Yangchuanosaurus*) of the skull length in the largest avetheropods belonging to the clade of Ceratosauria, Megalosauroidea, Allosauroidea and Tyrannosauroidea), 53% being the proportion of the maxilla in the closely related basal tetanurans *Sciurumimus* and *Monolophosaurus*. Following this tendency, we can estimate the skull length of *Torvosaurus* to approximately 115 cm (Fig. 4B), lower than what was proposed by Mateus et al. [4]. *Torvosaurus* was therefore not competing in size with the largest theropod *Tyrannosaurus* (maxilla length of ∼750 mm in CMNH 9380), *Carcharodontosaurus* (>710 mm in SGM Din-1) or *Giganotosaurus* (>680 mm in MUCPv-CH-1) but likely had a similar size than the tyrannosaurids *Daspletosaurus*, *Gorgosaurus* and *Tarbosaurus* [109] from the Cretaceous. Nonetheless, with a body length of around 10 meters (Fig. 4A) and a weight of approximately 4 to 5 tons (estimations based on [109]), *Torvosaurus gurneyi* represents the largest theropod from the Lourinhã Formation of Portugal, one of the largest land predators of the Jurassic, and the largest terrestrial predator discovered in Europe hitherto.

*Torvosaurus* occurrences are restricted to the Late Jurassic of Morrison and Lourinhã Formations, in United States and Portugal, respectively. The Portuguese form, *T. gurneyi*, is Late Kimmeridgian in age based on strontium and biostratigraphy [110]. The holotype specimen of *T. tanneri* is from Dry Mesa (Brushy Basin Member) which has been placed in the Late Kimmeridgian ([111]:1466), but the isotopic dates are still within the Early Tithonian in the new chronostratigraphic dates of ICS International Commission on Stratigraphy (www.stratigraphy.com) for the Late Jurassic.

The closest relative of the genus *Torvosaurus* is likely to be the European Bathonian *Megalosaurus* ([41], [61]), therefore the lineage leading to the genus likely originated during or around the Bathonian. At this time, the proto-Atlantic sea was well formed as demonstrated by ammonites and other sea fauna in the Portuguese west margin since the Early Jurassic. Therefore, the North American/European passage was already limited for terrestrial vertebrates well before the cladogenesis of *Megalosaurus*/*Torvosaurus* or *T. tanneri*/*T. gurneyi*. However, as suggested by Mateus et al. [21], the passage of *Torvosaurus* and other genera between North America and the Iberian Meseta may have happened during the temporary short-duration regional uplift around the Callovian/Oxfordian transition (ca. 163.5 Ma) that created the temporary opportunity of land gateways in the proto-Atlantic. The isolation of the Iberian block after that temporary uplift leaded to an important vicariance during nearly 10 My until the occurrence of many Laurasian taxa such as *Torvosaurus* in the Late Kimmeridgian. This pattern of occurrences shared by Morrison and Lourinhã Formations is also corroborated with the presence of other common genera, *Allosaurus*, *Ceratosaurus*, *Stegosaurus*, *Dryosaurus*, and related sister-taxa. This timing of vicariance also explains why the two regions have vertebrate faunas that are generically similar but specifically different. Finally, the true oceanization, with oceanic crust, of that section of North-Atlantic started during the Early Cretaceous.

### Other Occurrences of *Torvosaurus* in Portugal

#### Cranial bones

The anterior part of a right maxilla (ALT-SHN.116) discovered in the Lourinhã Formation of Praia da Corva (Torres Vedras) was described and referred to the taxon *Torvosaurus* sp. by Malafaia et al. [8]. The fragment of maxilla consists of an incomplete portion of the antorbital body and the anteroventral part of the ascending ramus [8]. The anterior part of the anterior ramus as well as the posterior portion of the antorbital body are missing, and both alveolar and dorsal margins of the antorbital body are strongly damaged. In lateral view, the anteriormost part of the lateral antorbital fossa has been preserved and is delimited by the antorbital ridge that bounds a depression filled with sediment laterally [8]. Two large maxillary alveolar foramina and one medium alveolar foramen are present at the level of what we interpret to be the fourth alveolus, just dorsal to the ventral margin of the maxilla. Only one small maxillary circumfenestra foramen seems to be preserved dorsal to the posteriormost alveolar foramen. As noted by Malafaia et al. [8], the lateral surface of the maxilla is smooth rather than rugose. In medial view, only the posterior part of the anteromedial process is preserved and displays two parallel ridges running longitudinally on the medial side of the process. The groove delimited by these two ridges is broad and shallow. The interdental plates are not well-preserved but are tall and clearly fused, and their surface seems to be striated by parallel grooves running ventrodorsally. The medial shelf is ventrodorsally broad but poorly protuberant, and its main axis is oriented posteroventrally. The nutrient groove is visible but not clearly marked, and two large nutrient foramina, likely of the second and fourth alveoli, are present at the level of this groove. Dorsomedially, the nasal contact is broad and not visible laterally [8]. An unerupted tooth can be seen throughout a small fracture and its mesial carina bears around 10 denticles per 5 mm [8].

This fragment of maxilla was assigned to *Torvosaurus* sp. by Malafaia et al. [8] based on the absence of a maxillary fenestra between the antorbital fenestra and the nasal contact, and the shape and position of the antorbital ridge bounding the antorbital fenestra anteroventrally [8]. We agree with the assignment of this fragment to *Torvosaurus* but for different reasons. The presence (or absence) of a maxillary fenestra/fossa cannot be determined due to the fact that most of the anterior corner of the lateral antorbital fossa and the posteroventral portion of the ascending process are not preserved. The maxillary sinus are always located in this area in all basal theropods, including *Torvosaurus*, and the presence of a maxillary fenestra cannot therefore be ruled out. In fact, the shallow maxillary fossa diagnostic of *Torvosaurus tanneri* [27] is not preserved in ALT-SHN.116, and what has been interpreted as the anteriormost rim of the antorbital fenestra by Malafaia et al. ([8]:fig. 2B1) is, in fact, a diagnostic ridge located on the anteriormost corner of the lateral antorbital fossa (pers. obs.). Likewise, an antorbital ridge forming a lateral rim that bounds a recess within the anteriormost corner of the lateral antorbital fossa is a feature shared by a few basal theropods such as *Ceratosaurus*, *Torvosaurus*, *Afrovenator* and *Dubreuillosaurus* (pers. obs.). As correctly noticed by Malafaia et al. [8], an antorbital ridge located just below the antorbital fenestra in the anteroventral part of the lateral antorbital fossa is indeed present in *Torvosaurus*, but equally shared by abelisaurids, *Monolophosaurus* and *Eustreptospondylus* (pers. obs.). However, the rim of the antorbital fenestra is not preserved in ALT-SHN.116, and the position of the antorbital ridge relative to the antorbital fenestra cannot therefore be used as a diagnostic feature. Nonetheless, this fragment of maxilla includes several important features that support affinities with the genus *Torvosaurus*. ALT-SHN.116 belongs to tetanurans given the presence of a moderately (or strongly) elongated anterior ramus, to Megalosauria or Allosauria (as proposed by [61]) due to the position of the anteromedial process, immediately ventral to the dorsal surface of the anterior ramus [61], to the clade including *Torvosaurus* and *Megalosaurus* because of the tall interdental plates and to *Torvosaurus* by the presence of fully fused interdental plates [61] and a prominent anteroposteriorly oriented ridge (different from the antorbital ridge) in the anteriormost corner of the lateral antorbital fossa, an autapomorphy of *Torvosaurus* (pers. obs.). ALT-SHN.116 can be even assigned to the new taxon *Torvosaurus gurneyi* by the presence of interdental plates extending to the same level than the lateral wall, the straight ventral margin of interdental plates (absence of V-shape interdental plates), two longitudinal ridges of the anteromedial process that get flared at the same level posteriorly, and the absence of a prominent ridge posterior to the anteromedial process. ALT-SHN.116 can therefore be referred to *Torvosaurus gurneyi* with confidence.

A mesialmost tooth (ML 962) from the Early Tithonian of the Lourinhã Formation in Praia da Area Branca (Lourinhã) was recently identify to belong to *Torvosaurus tanneri* [6] based on size (CH >80 mm), crown elongation (CHR of 2.7), large denticles (∼8 denticles per 5 mm on both carinae), outline of the cross-section (CBR of 0.64) and position and basal extension of the mesial carina ([6]: fig.9). Given the fact that ML 962 and *T. gurneyi* have close paleogeographical and stratigraphical distributions, we tentatively assign the tooth to the Portuguese species of *Torvosaurus*.

A large tooth (FUB PB Ther 1) discovered in the Late Kimmeridgian of the Lourinhã Formation (Sobral Member = Praia Azul Member *sensu* [112]) in Porto das Barcas was ascribed to an indeterminate Carnosauria by Rauhut & Kriwet [113] based of large size and interdenticular sulci. A discriminant analysis used by [114] classified it as *Ceratosaurus* although the authors admitted that this analysis “cannot provide a genus-level classification for a tooth that came from a taxon for which there are no data in the standard” ([114], p. 715). A better understanding of theropod dentition, as well as morphometric data collected in the dentition of *Torvosaurus*, allows us to confidently refer this lateral tooth to this taxon, and tentatively to *T. gurneyi* given the stratigraphic and geographic contexts. Indeed, FUB PB Ther 1 shares a combination of features only seen in *Torvosaurus* lateral teeth such as a large (CH = 80 mm) moderately labiolingually compressed (CBR of 0.53) crown bearing large and coarse denticles (6.5 denticles/5 mm on both carinae), well-developed interdenticular sulci, a clearly-visible braided enamel texture, and a mesial carina centrally positioned on the crown (not offset or twisted) and extending on the apical half of the crown. Large teeth of eight centimetres or more are only borne by ceratosaurids, non-coelurosaur tetanurans and tyrannosauroids, and lateral teeth with very large denticles (<8 denticles/5 mm) by Megalosauridae, Carcharodontosaurinae, and Tyrannosauridae (pers. obs.). Tyrannosaurid teeth are distinctly incrassate (CBR>0.55), and the mesial carina of carcharodontosaurine and *Ceratosaurus* teeth either reaches the cervix or extend just above it (pers. obs.). Among Megalosauridae, large crowns with very well-developed interdenticular sulci and marked enamel texture are, to our knowledge, a combination of features only existing in *Torvosaurus*. Furthermore, the latter is the only megalosaurid theropod from the Late Jurassic of Portugal.

#### Postcranial bones

**Figure 11:**
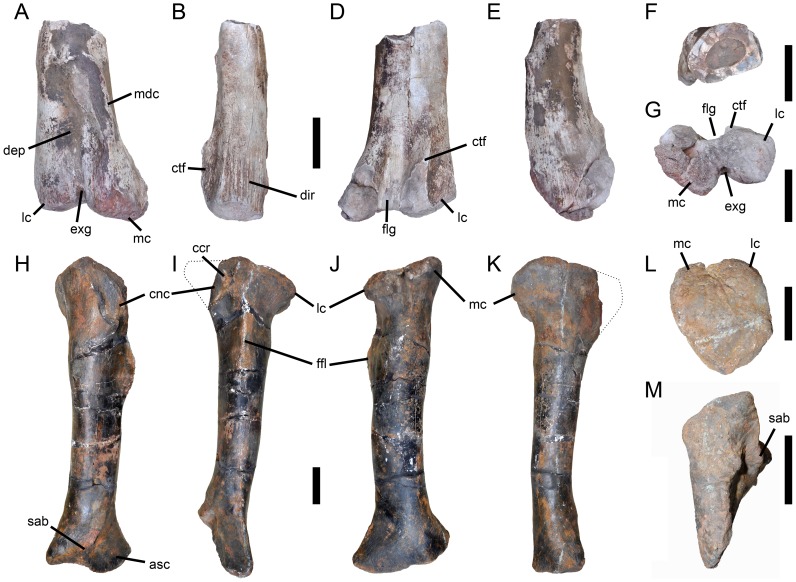
Femur and tibia of Megalosauridae from the Late Jurassic of Portugal. Distal portion of a left femur (ML 632) of a megalosaurid tentatively referred to *Torvosaurus gurneyi* in **A**, anterior; **B**, lateral; **C**, posterior; **D**, medial; **E**, proximal; and **F**, distal views. Incomplete left tibia (ML 430) of *Torvosaurus* sp. (and tentatively referred to *Torvosaurus gurneyi*), with reconstruction of missing part of cnemial crest, in **A**, anterior; **B**, lateral; **C**, posterior; **D**, medial; **E**, proximal; and **F**, distal views. Abbreviations: **asc**, contact with astragalus; **ccr**, cnemial crest ridge; **cnc**, basal part of cnemial crest; **ctf**, crista tibiofibularis; **dep**, anterodistal depression; **dir**, distal ridges; **exg**, extensor groove; **ffl**, fibular flange; **flg**, flexor groove; **lc**, lateral condyle; **mc**, medial condyle; **mdc**, medio-distal crest; **sab**, supracetabular buttress. Scale bars = 10 cm.

The distal portion of a right femur (ML 632; Fig. 11A–G) from Cadaval (Quinta do Gradil) has been briefly reported by [4] and tentatively assigned to *Torvosaurus* based on its large size. The femur preserves the distal diaphysis, which includes two partially damaged condyles, and a portion of the shaft is preserved to the proximal extension of the mesiodistal crest. The bone is massive, the proximo-distal length of the distal portion measuring more than 370 mm (Table 5), and one can estimate the total length of the whole bone to around 1110 mm based on the length and proportion of the femur of *Megalosaurus bucklandii* (BMNH 31806; [41]). The minimum circumference of the shaft is 370 mm at the level of the break and its transversal ratio (lateromedial width/anteroposterior width) is 1.44.

**Table 5:** Measurements of limb bones tentatively referred to *Torvosaurus gurneyi*.

	Measurements (mm)
**Femur (ML 632)**	
Maximal length of distal portion	370
Minimal circumference	390
Maximal circumference	600
Anteroposterior diameter of distal diaphysis	110
Lateromedial diameter of distal diaphysis	235
**Tibia (ML 430)**	
Maximum length	820
Minimum circumference	385
Circumference at the level of the fibular crest	470
Fibular crest length	140
Anteroposterior diameter of proximal diaphysis	110
Lateromedial diameter of proximal diaphysis	290
Anteroposterior diameter of distal diaphysis	240
Lateromedial diameter of distal diaphysis	180

The shaft expands mediolaterally towards the distal diaphysis and gives rise to two condyles separated by the extensor groove anteriorly (Fig. 11A) and the flexor groove posteriorly (Fig. 11D). The medial condyle is anteroposteriorly longer than the lateral one and elliptical in outline in distal view (160 mm long by 80 mm wide at its midpoint; Table 5) with its long axis directed posterolaterally (Fig. 11G). Fragments of the laterodistal, mesiodistal and posterior surface of the medial condyle are missing so that it is not possible to know the proximal extension of the articulating surface posteriorly. This surface is, however, well-preserved anteriorly and extends further proximally than in the lateral condyle. The medial margin of the medial condyle corresponds to a planar surface bearing a shallow concavity centrally positioned on its distal most part. The medial margin of the diaphysis displays many shallow striations extending along 190 mm of the medial side of the femur posteriorly (Fig. 11E). The posterior margin of the medial condyle is strongly convex, forming a large protuberance delimiting the flexor groove medially. The latter is lateromedially large (40 mm width) and extends along 120 mm of the bone surface, keeping the same width proximo-distally. The floor of the flexor groove is flat and grooved on its medial part, and the larger sulcus penetrates the bone on the proximomedial corner of the flexor groove.

The lateral condyle is roughly D-shaped (100 mm anteroposterioly by 105 mm lateromedially), with the concavity facing anteriorly (Fig. 11G). The condyle projects posteromedially to form the crista tibiofibularis in which most of the posterior portion is missing (Fig. 11B). The crista tibiofibularis corresponds to a large crest of 55 mm in width in its posteriormost part, and extends proximo-distally along 130 mm of the lateral surface of the femur. The crest tapers proximally and curves proximo-laterally so that the medial margin of the crista tibiofibularis is convex whereas the lateral surface is weakly concave. This surface is also deeply striated by radiating grooves converging proximally. The posterior margin of the lateral condyle is shallowly concave and weakly grooved, in contrast to its lateral and anterior surfaces which, together, form a wide convexity. The latter is covered by large, deep and well-developed parallel striations in which the longest extend along 65 mm of the bone. The largest and deepest grooves are located on the anterolateral margins of the lateral condyles and show a strong attachment of the disto-femoral muscles (Fig. 11B).

The anterodistal surface of the femur is deeply excavated by the extensor groove which is narrower and deeper than the flexor groove (Fig. 11A). The extensor groove corresponds to a shallow depression in its proximal part and a deep fossa more distally, having a width of 20 mm in its distalmost part. The anterior surface of the femur displays a massive medial distal crest can-shaped in outline (or a reversed J) and extending from above the medial condyle along 190 mm of the shaft (Fig. 11A). The medial distal crest is poorly delimited laterally and bounded by a short elevation of the shaft proximally. A wide depression, bounded by the medial distal crest laterally, occupies the central part of the anterodistal surface of the femur. This large concavity has a rugose surface proximo-laterally, just medial to the medio-distal crest, and merges with the extensor groove distally (Fig. 11A).

The femur is tentatively referred to *Torvosaurus gurneyi* based on its size, paleogeographic and stratigraphic distributions, and a combination of features only seen in derived megalosaurids. ML 632 belongs to Orionides based on the presence of an extensor groove anteriorly, to Megalosauroidea due to the absence of a large depression on the anterodistal surface of the mesial condyle of the distal diaphysis (the rugose depression that exists on the anterior surface of the medial condyle in coelophysoids, ceratosaurs and allosauroids cannot be confused with the centrally positioned depression on the anterodistal surface of ML 632), and to Megalosauria based on the longitudinal and narrow tibiofibularis crest [61]. Among Megalosauria, the protuberant medial distal crest running on the anterodistal surface of the femur is absent in Spinosauridae and some megalosaurids such as *Eustreptospondylus* and *Leshansaurus*. It is poorly developed in *Megalosaurus* [41], and only well-developed and protuberant in the femur TATE 0012 ([95]:fig. 16B), a specimen of large megalosaurid of the Morrison Formation [95] referred to *Torvosaurus tanneri* [61]. Furthermore, the distal diaphysis bears large and deep striations on its anterior, lateral and medial surface, a condition shared with TATE 0012 ([92]:fig. 16). As for TATE 0012, ML 632 is tentatively assigned to *Torvosaurus*. It however differs from TATE 0012 by a long axis of the medial condyle directed posterolateral in distal view (a condition shared with Baryonychinae [61]), a much deeper extensor groove, and a long axis of the medial distal crest directed proximo-distally (like in *Megalosaurus*) rather than proximo-laterally ([95]:fig. 16B). In fact, a posterolateral orientation of the mediodistal condyle as well as a prominent medial distal crest curving proximo-laterally and delimiting a large concavity medially, seem to be two autapomorphies of ML 632 among Megalosauridae (pers. obs.). The femur comes from a different site than the type specimen of *T. gurneyi* and cannot be assigned to this taxon as the latter did not preserve any limb bones. Nevertheless, given the geographic and stratigraphic position of ML 632 and the numerous features shared with TATE 0012, it is likely that the bone belongs to *Torvosaurus gurneyi*. However, this referral has to be regarded as tentative, pending detailed description and analysis of TATE 0012.

With a lateromedial width of 235 mm for the distal diaphysis and an approximate length of 1100 mm for the femur (Table 5), ML 632 pertains to an animal of around 3 to 4 tons, for a body length of around 10 meters (estimations based on [109] and [115]).

A large sized left tibia (ML 430; Fig. 11H–M) from Casal do Bicho was the first bone unequivocally ascribed to *Torvosaurus* in Portugal [7]. The tibia has a unique character combination that allows a generic identification, as recognized since Britt [27], including the high diaphyseal perimeter/length ratio, low astragalar contact surface and short and round cnemial crest. With a total length of 820 mm and a minimum circumference of 385 mm (Table 5), ML 430 pertained to a slightly bigger animal than BYU VP 2016 (length of 725 mm, min. circ. of 327 [27]) that should have had a body mass of around 1.6 to 1.7 tons for a body length of around 7 meters (estimation based on [109] and [115]). Given its paleogeographic and stratigraphic distributions and a combination of features only existing in *Torvosaurus* tibiae, ML 430 is tentatively referred to *Torvosaurus gurneyi*.

#### Tracks

Only in rare cases in vertebrate paleontology, one can establish a connection between a track and a genus of species of trackmaker. However, in Portugal there are no other theropod that could rivalize *Torvosaurus* in size, and produce such large tracks as the ones from the beds of Porto Dinheiro (ML 2035, [116]:fig. 9). Being 79 cm long and 60 cm wide, ML 2035 is one of the largest theropod tracks known from the Jurassic. These tracks found at the base of Sobral Member of Lourinhã Formation are dated as Late Kimmeridgian, just as the *Torvosaurus* bones from Portugal. Nevertheless, due to the absence of clear pedal autapomorphies that are recognizable in *Torvosaurus* tracks, the trackmaker of ML 2035 is tentatively referred to *Torvosaurus*.

#### Embryos

Araújo et al. [9] recently reported an incomplete right maxilla and dentary and three centra of a single or several *Torvosaurus* embryos (ML 1188:fig. 9A–F) from the Late Kimmeridgian Lourinhã Formation (Sobral Member that overlies Porto Novo-Amoreira Member from which *T. gurneyi* type comes from) of Porto das Barcas. The cranial and postcranial elements are referred to *Torvosaurus* sp. based on the absence of both medial antorbital fossa and medial pneumatic complex on the maxilla, tall interdental plates and blunt anterior margin of the dentary, low angle of the ascending ramus and the tongue-shaped posterior extremity of the jugal ramus [9]. As highlighted by these authors, some notable difference can however be observed in between the maxilla of the embryonic and adult specimens of *Torvosaurus*, the most important being a short anterior ramus, unserrated crowns and unfused interdental plates, all interpreted as ontogenetic features. There are four preserved interdental plates for the maxilla (*contra* Araújo et al. [9]), a first one situated between the first and second maxillary teeth, a second incomplete one between teeth 2 and 3 ([9]:fig. 9D), a badly preserved one between teeth 3 and 5 and an isolated one below the maxilla (pers. obs.). As observed in the interdental plates of the dentary, and similar to the condition seen in the adult *Torvosaurus gurneyi* and *Megalosaurus bucklandi*, the maxillary interdental plates are tall and all have a vertical rectangular outline. The lateral wall of the maxilla is not visible in the embryos ML 1188 and it is unknown whether the interdental plates were extending at the same level than the ventral margin of the lateral wall like in *T. gurneyi*. Nonetheless, the ventral margins of the plates are straight and do not display the “V-shaped” condition shared by the American taxon. Likewise, there is no apparent ridge posterior to the anteromedial process, as seen in *T. tanneri*. Nevertheless, these features may all vary ontogenetically in theropods so that ML 1188 is tentatively assign to the species *T. gurneyi* based on paleogeographical and stratigraphical contexts only.

## Supporting Information

File S1 **Morphological variations in the interdental plates of** ***Dilophosaurus wetherilli*** **.**
(PDF)

Table S1 **Morphology of interdental plates in non-maniraptoriforms theropods.**
(PDF)

Text S1 **Institutional abbreviations, character list and datamatrix.**
(PDF)
