# Supplementary material for: Torvosaurus gurneyi n. sp., the Largest Terrestrial Predator from Europe, and a Proposed Terminology of the Maxilla Anatomy in Nonavian Theropods
Source: PLoS One. 2014 Mar 5;9(3):e88905. doi: 10.1371/journal.pone.0088905 (PMC3943790; doi:10.1371/journal.pone.0088905)
Supplement: File S1 — Morphological variations in the interdental plates of Dilophosaurus wetherilli . (PDF) [file pone.0088905.s001.pdf]

Supplementary Information File S1

Torvosaurus gurneyi n. sp., the largest terrestrial predator from Europe, and a proposed terminology of the maxilla anatomy in nonavian theropods

Christophe Hendrickx and Octávio Mateus

Morphological variations in the interdental plates of *Dilophosaurus wetherilli*

Two distinct morphotypes of the interdental plates can be seen in the specimens referred to the taxon *Dilophosaurus wetherilli*. The holotype UCMP 37302 and the paratype UCMP 37303, both juvenile individuals, possess fully fused and subrectangular interdental plates with an anteroposterior axis of elongation and straight ventral margins of the plates. On the other hand, the juvenile specimen TMM 43646-1 and the adult individual UCMP 77270 display separated interdental plates that are subquadrangular to subrectangular, with a long axis directed ventrodorsally and strongly "V-shaped" ventral margins ([42], pers. obs.). As far as we know, such variability of interdental plates is unique among theropods and cannot be explained by ontogeny, therefore the existence of two taxa of *Dilophosaurus* in the Kayenta Formation, as previously suggested by [83], seems highly plausible to us. In fact, a second species of *Dilophosaurus*, *D. "breedorum"* was named by Welles and Pickering [117] based on the specimen UCMP 77270 in a controversial paper, but the name does not follow the condition of the ICZN to be recognized as valid (see http://theropoddatabase.blogspot.com.ar/2010/05/pickerings-taxa-6-dilophosaurus.html for more information). UCMP 77270 consists of an incomplete skull and skeleton discovered in 1964 that was initially referred to a larger specimen of *D. wetherilli* [118] then thought to be a new genus closely related to *D. wetherilli* [83]. Despite the fact that some differences between UCMP 77270 and the type specimens were noted by Welles and Pickering [117], and later by Tykoski [42] and Irmis [119], the scientific literature only recognizes the existence of one species of *Dilophosaurus* from the Kayenta Formation hitherto (e.g., [42,47,53,62,119,120]).

The stratigraphic level from which the type and paratype specimens came, as well as UCMP 77270 collected in Moenkopi Wash, does not seem to be well established, and there is

some potential uncertainty with correlating the exact position of UCMP 77270 with those from Gold Spring like TMM 43646-1 (Tykoski, pers. comm.). According to Welles [118,121], the type locality is listed at an elevation of 1402 meters near the base of the Kayenta Formation, slightly above the contact with the Moenave Formation and about 38 meters (126 feet) above the top of the Chinle Formation. However, the Texas *Dilophosaurus* quarry is actually closer to 1550 meters in elevation and clearly in the middle third of the Kayenta Formation, certainly not near the contact with the Moenave Formation. This is also the case for TMM 43646-1 that was discovered in the middle third of the Kayenta Formation near Gold Spring (Tykoski, pers. comm.). Although no one has been able to work out the stratigraphic separation between the type specimen, UCMP 77270 and TMM 43646-1, and the amount of time separating them, it may be possible that there is enough time represented in the section to capture evolutionary change to the species level (Tykoski, pers. comm.).

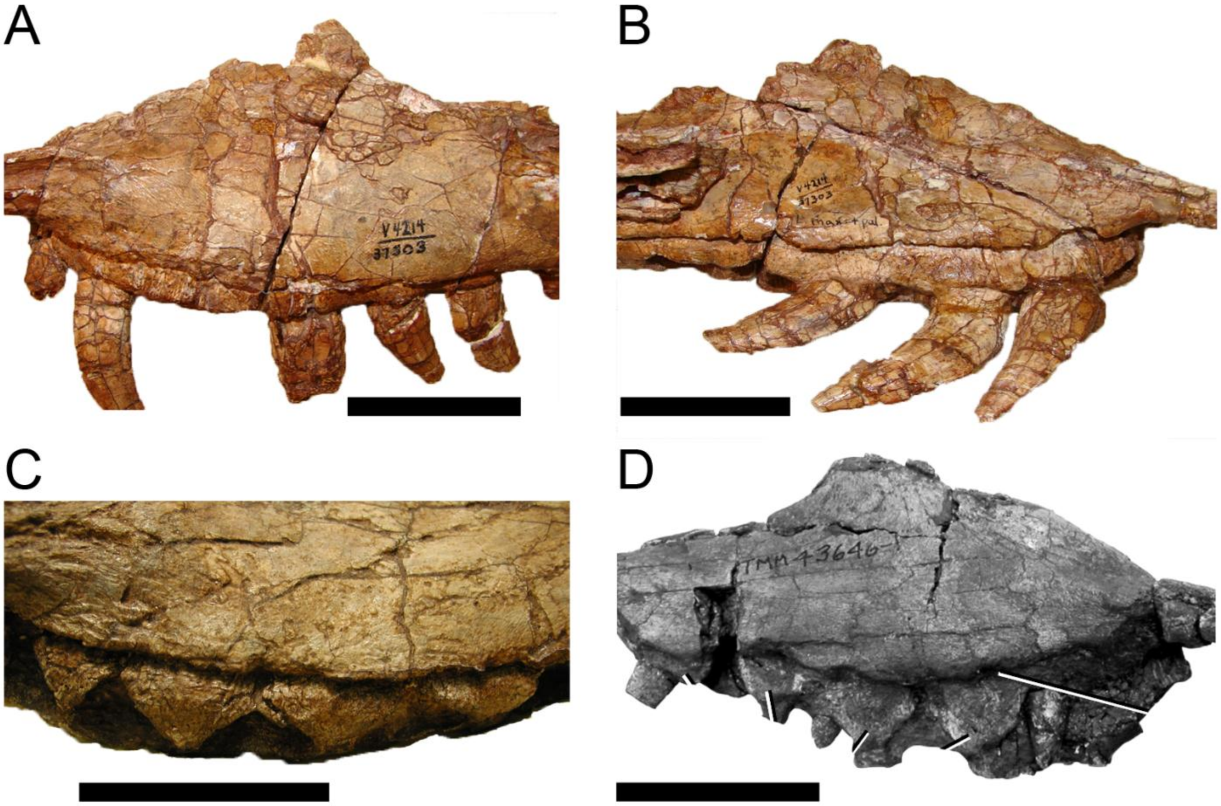

Figure S1. Morphological variations in the interdental plates of *Dilophosaurus*. A-B, Right (**A**) and left (**B**) maxillae of the juvenile specimen UCMP 37303 (paratype) of *Dilophosaurus*

wetherilli in medial views representing morphotype I of interdental plates (courtesy of Martín Ezcurra). **C-D**, Interdental plates of left maxilla (**C**) of adult specimen UCMP 77270 referred to *Dilophosaurus "breedorum"* by [117] (courtesy of Ronald Tykoski), and partial left maxilla (**D**) of juvenile specimen TMM 43646-1 (from Tykoski [42]:figure 36B) referred to *Dilophosaurus wetherilli* in medial views and representing morphotype II of interdental plates. Scale bars = 5 cm.

Given the important difference in the interdental plates between the type specimens and UCMP 77270 and TMM 43646-1, accompanied by difference in the maxillary and dentary tooth count and, in UCMP 77270, the participation of the prefrontal in the nasal crest, the presence of a groove along the posteroventral edge of the postorbital, a posterolateral sulcus in the quadratojugal [117], and closed dorsal and proximal caudal neurovascular suture [117], all features that seem to be autapomorphies, we tentatively accept the existence of two species of *Dilophosaurus* in the Kayenta Formation, pending on a thorough description of UCMP 77270 and TMM 43646-1 to support the creation of a new species of *Dilophosaurus*.

117. Welles SP, Pickering D (1995) An extract from: Archosauromorpha: cladistics and osteologies. A Fractal Scaling in Dinosaurology Project. 70 p.

118. Welles SP (1970) *Dilophosaurus* (Reptilia, Saurischia), a new name for a dinosaur. Journal of Paleontology 44: 989.

119. Irmis RB (2007) Axial skeleton ontogeny in the Parasuchia (Archosauria: Pseudosuchia) and its implications for ontogenetic determination in archosaurs. Journal of Vertebrate Paleontology 27: 350–361. doi:10.1671/0272-4634(2007)27[350:ASOITP]2.0.CO;2.

120. Gay R (2005) Sexual dimorphism in the Early Jurassic theropod dinosaur *Dilophosaurus* and a comparison with other relataed forms. In: Carpenter K, editor. The Carnivorous Dinosaurs. Bloomington, Indiana: Indiana University Press. pp. 277–283.

121. Welles SP (1954) New Jurassic Dinosaur from the Kayenta Formation of Arizona. Geological Society of America Bulletin 65: 591–598. doi:10.1130/0016-7606(1954)65[591:NJDFTK]2.0.CO;2.
