# Supplementary material for: Torvosaurus gurneyi n. sp., the Largest Terrestrial Predator from Europe, and a Proposed Terminology of the Maxilla Anatomy in Nonavian Theropods
Source: PLoS One. 2014 Mar 5;9(3):e88905. doi: 10.1371/journal.pone.0088905 (PMC3943790; doi:10.1371/journal.pone.0088905)
Supplement: Table S1 — Morphology of interdental plates in non-maniraptoriforms theropods. (PDF) [file pone.0088905.s002.pdf]

Supplementary Information Table S1

***Torvosaurus gurneyi* n. sp., the largest terrestrial predator from Europe, and a proposed terminology of the maxilla anatomy in nonavian theropods**

Christophe Hendrickx and Octávio Mateus

Morphology of interdental plates in non-maniraptoriforms theropods. Fusion of interdental plates: fused (0), separated (1); ventral extension of interdental plates relative to lateral wall of maxilla: fall short (0), well dorsal (1); Shape of interdental plates: rectangular with a straight ventral margin (0), subpentagonal with a V-shaped ventral margin (1); triangular or trapezoidal (2).

Taxa	Fusion	Ventral extension	Shape
Abelisaurus comahuensis	0	0	0
Acrocanthosaurus atokensis	0	0	0
Afrovenator abakensis	1	0	1
Albertosaurus sarcophagus	1	1	1
Alioramus altai	1	0	1
Allosaurus fragilis	0	1	1
Aucasaurus garridoi	0	0	0
Baryonyx walkeri	1	0	1
Carcharodontosaurus iguidensis	0	0	1
Carcharodontosaurus saharicus	0	0	0
Ceratosaurus nasicornis	0	0	0
Coelophysis rhodesiensis	1	0	2
Compsognathus longipes	1	0	0
Daspletosaurus torosus	1	1	1
Dilophosaurus wetherilli	0	0	0
*Dilophosaurus "breedorum"**	1	0	1
Dubreuillosaurus valesdunensis	1	0	1
Duriavenator hesperis	1	0	1

Eocarcharia dinops	0	0	1
Eodromaeus murphi	1	0	0
Eotyrannus lengi	0	0	1
Eustreptospondylus oxoniensis	1	0	1
Frenguellisaurus ischigualastensis	0	0	0
Genyodectes serus	0	0	0
Giganotosaurus carolinii	0	0	0
Gorgosaurus libratus	1	0	1
Guanlong wucaii	1	1	1
Indosuchus raptorius	0	0	1
Kileskus aristotocus	1	0	1
Kryptops palaïos	0	0	0
Leshansaurus qianweiensis	1	0	?
Majungasaurus crenatissimus	0	0	0
Mapusaurus roseae	0	0	1
Marshosaurus bicentesimus	1	0	1
Masiakasaurus knopfleri	0	0	1
Megalosaurus bucklandii	1	1	0
Neovenator salerii	0	1	1
Noasaurus leali	0	0	0
Piatnitzkysaurus floresi	1	0	1
Proceratosaurus bradleyi	1	0	1
Raptorex kriegsteini	1	0	1
Rugops primus	0	0	1
Scipionyx samnicicus	1	0	0
Shaochilong maortuensis	0	0	0
Sinosaurus triassicus	1	0	1
Sinraptor dongi	1	0	1
Spinosaurus aegyptiacus	1	0	2
Suchomimus tenerensis	1	0	1
Tarbosaurus bataar	1	1	1

Torvosaurus gurneyi	**0**	**0**	**0**
Torvosaurus tanneri	0	1	1
Tyrannosaurus rex	1	1	1
Zuolong sallei	1	0	1

* specimens UCMP 77270 and TMM 43646-1
