# Supplementary material for: Torvosaurus gurneyi n. sp., the Largest Terrestrial Predator from Europe, and a Proposed Terminology of the Maxilla Anatomy in Nonavian Theropods
Source: PLoS One. 2014 Mar 5;9(3):e88905. doi: 10.1371/journal.pone.0088905 (PMC3943790; doi:10.1371/journal.pone.0088905)
Supplement: Text S1 — Institutional abbreviations, character list and datamatrix. (PDF) [file pone.0088905.s003.pdf]

Supplementary Information Text S1

Torvosaurus gurneyi n. sp., the largest terrestrial predator from Europe, and a proposed terminology of the maxilla anatomy in nonavian theropods

Christophe Hendrickx and Octávio Mateus

Institutional abbreviations

ALT–SHN, Laboratorio de la Associação Leonel Trindade, Sociedade de História Natural Sociedade de História Natural, Torres Vedras, Portugal; AMNH, American Museum of Natural History, New York, USA; BHI, Black Hills Institute, Hill City, South Dakota, USA; BMNH, Natural History Museum, London, UK; BYU-VP, Brigham Young University Vertebrate Paleontology, Provo, Utah, USA; CMNH, Carnegie Museum of Natural History, Pittsburgh, USA; CV, Chongqing Museum of Natural History, Chongqing, China, FMNH, Field Museum of Natural History, Chicago, USA; FUB PB, Freie Universität Berlin, Berlin, Germany; IGM, Institute of Geology, Ulaan Baatar, Mongolia; IVPP, Institute for Vertebrate Paleontology and Paleoanthropology, Beijing, China; KMV, Kunming Municipal Museum, Guandu district, China; LACM, Los Angeles County Museum of Natural History, Los Angeles, California, USA; MCF-PVPH, Museo Municipal 'Carmen Fuñes', Plaza Huincul, Argentina; ML, Museu da Lourinhã, Lourinhã, Portugal; MNN, Musée National du Niger, Niamey, Niger; MUCPv-PH, Museo de Ciencias Naturales de la Universidad Nacional de Comahue, Lago Barreales, Argentina; MWC, Museum of Western Colorado, Fruita, USA; OUMNH, Oxford University Museum, Oxford, UK; PVL, Fundación "Miguel Lillo", San Miguel de Tucumán, Argentina; SGM, Ministère de l'Énergie et des Mines, Rabat, Morocco; TATE, Tate Museum, Casper College, Casper, USA; TMM, Texas Memorial Museum, Austin, Texas; USA; UCMP, University of California Museum of Paleontology, Berkeley; UMNH, Natural History Museum of Utah, University of Utah, Salt Lake City, USA; USNM VP, United State National Museum Vertebrate Paleontology, Washington, District of Columbia, USA; ZPAL, Institute of Palaeobiology of the Polish Academy of Sciences, Warsaw, Poland.

Character list

Characters 1-351: from [61], with the modification of character 26. Character 352 and 353 were created and coded for each taxa.

*26. **Maxilla**, development of maxillary 'fenestra': absent (0), deep and well-delimited fossa (1), shallow and poorly delimited fossa (2), fenestra (3).*

*352. **Maxilla**, shape of interdental plates (separated or fused): rectangular (0), subpentagonal (1), triangular or trapezoidal (2).*

*353. **Maxilla**, neurovascular opening on the dorso-medial edge of the jugal ramus: absent (0), present as one foramen (1), present as several foramina (2).*

Data matrix

nstates 5

xread

353 62

Eoraptor

00010000?00000??????0101000?0?0010000?0000100??0001?000000100000?0000000000?10?00??0?001000?0000?0??????0??00?0?101000000?0?0000?00?0000?0?00?000100000000000-0?????????-0000000000000?0?00000000000000?0000001?0010000?000001000000101000000000?00100000100000000000010000000?00000000000??000?0000000?00000?0000?000?000000000???-0000000000000000000000000??

Herrerasaurus

00001000000000????0?0001000?0?0?00000?0000100??00120100000000000?00000100000000020?000010?00000?10??00000000?000000?10000?0?0000?000010010000?0?0100000000000-00?000010?-0000000000000?0?1000000?0000000000000??001??00?1?011?0??00010100000010000010100010000000000001 0000000?00000000000?0?001000000?00000??0000100000000000001??-010000100000000000000000000

Acrocanthosaurus

020?1100?00000011110000003111111100010?000000211101211100020212011?1121111020110?02??110121000001001101000120111111?111002100101020010?11111?000100010000010011012?00110011?00?0012110001000110011211110?0021000000101?1?110110120012001210?001011110000113110111111000????010?10??1110110201201?120?201111000220111011110000110010312?1010?1110?1??01210111001?100

Aerosteon

??01?000?111??????????10000200???110121???????11?01001?1?00010001011010111?????2?110??0?????????0101100121????????????????????????????????01011010010221110110201201?120??????????????????????????1001?412????01112021110??????????????

Afrovenator

?????????0010101100000100120011000?????0?000201001?011001011000?????????00?001111001??????
??0000000001?????10?11001?0?011000010001101100001?001?0
00001???????100000???1?01010??????????0121?????00?0?????0?31001111?100001?10?01101011?011?2012?1?0
0?001011100011101100001100001000021?11010110???111011?011100??110

Allosaurus

00101[01]000001000101010000031111110001101100002[01]2[01]0010110000000101111100011020000
002001101100011010010010201201111111112000100110201111111110000100011020010011001100110010[01]
100001011000100001000101111000011?0000000101?1101101200120012101011000010001113100111111000011
1010010211110110201201?1100200111000[12]1011101111000011001021221010111102111012101110010110

Angaturama

11???201?1121?11???0??????????????0??
????????????????????????1?0??????????????????110111701311??
??
??

Australovenator

???
?????????????????????????00001????????????100001000110??
?????????0?????????????????????????????00010101????01?1111??101??220
1210111100011100114122101011121211101211????010???

Baryonyx

111?0201011210110?0000???02???1??01????0?001202011?0??????0??0?1111100??????0?110110100010
11?01??010210?011??????????001101010?[12]101???1??0100101100013001011100[12]?10010010010001101111
10100011000101010?0???000?00??1??1?00??0?1?0100012111100010011010????????????10?0?11010?101?11101
10[01]0?2?1?0????0011000????1????00110100????????110?0??????????????0?????????1?

Carcharodontosaurus

??????0??000000111100001031101110101111000?02?1?0??111100??21211211?211????0????????110121
?0??0100??01121121???1???????2?10???????????????1000020000?????10?110?2??????0??01?0?00101?0???1????0?
?????????????100?0???1???1??1??1???????11?????0?????0??1
1?????22??1101111??01?100???????010??????????????????????[01]0

Ceratosaurus

021010001000000000000000010100010120?0000002020011000000010000111111000101001001000101
0100000000110100001110000?100010000001??1000000100100010000010100100011021?0100010000000021100
000010100001010211??10000210?0100001101011110100100110000????0??1??1?01?00010?010?11100?0000111
011110110100001[01]0001001100110[01]001102100010100200211101001021??1?0001??10???00

Chilantaisaurus

???
???

????????????????????????????0120110000?????????????????????11??????10????2110???????????????????????????22??111???1000?1100??41221????1?????????2101??0??????

Chuandongocoelurus

??0????????????????????0??????????????0?????????0???00???0011101011???1?0?????0?????????????00?101000100000?1000041?2121011???21110?100??100?????

Coelophysis_bauri

00101211000010????0?011010100?0000000?0000101??0000000000000000?0?00000110110100000?0000000?0?2??1????0??1?0?0?0?00??00110?0?0000?00?000000010000?000000001100?100011000?000001011000000?000000000100000000011?1100?01?0000010020001010000000?0[01]?00101110101000000100100011000011011110100?10000001000010000110[01]00?00?000?010001100021000000101111000110000000?0

Coelophysis_rhodesiensis

00???211000010???00?01101100?00000000?020010100001[01]00000000?000100000000?0110?0000000000000000200100?000?1000000000100010000000000000000010000000010000000011011?0001100000000010110000000?0000000010000?0000?11?1100001?000001002000101000?0010[01]000110110101000000100100011000111011110000?1000?011100010000111[01]001102000?0100?11000210000001011110001101000020

Compsognathus

00???100000100????0?010003100?1??0000?0000000??00000?000?00?00????1?00????????0??0????0??00?????0??0?0??????1?1????1?0000?00??0??100??10100?0000?0??0000100?100[12]???????100?001??001100?000????0000??1??0??000?0?00?1?0?0111?011100?0??[12]??0???0???0??01?1310011????0?000??010?11??110011?20?201?011?11?10??002???111?????????1?0???????01011???????01??0?11?01010?

Concavenator

?????0???????0??????001003??0?1?00?1111?10102010000?110??10??2????1??1020?0??????[01]0??100?????????????????[12]?1??10????1???0211?1?1??[01]100?000????1?111110?10?1??0?1??10??0?1010001??????????????0?0?0?10?11??111011020????????02011???1?22??1100??????????????[12]2??0?0?1?1?????0???0?1?00?????

Condorraptor

???0010001????????01001????????0000?00101?00001000?2001?011101001?0002???01??101???0?1??01?01010????????0????0???00001100????????0??01000001?00??11????????????????1?01???0?????

Cryolophosaurus

???????????????????00?????????1??00?1001?01010?000?0111??0?00[01]0?1?10000?10??000000??10001000001??1?0010????0??????1??0????????????10?000000000?0?01??????0?01000????????00?0?000?0?1000??001000??010?0?????000200????0?01???????????????????????0?10001??????????????00???00???0?????011??0????0???0?0??10011001?0?0011010000000?2??????01001011??0??????????????

Dilophosaurus

01?11211000010000000001000100000000?100200100??00?200100001?0001001000?1101101000000000
?000000200101000001??0??0?????201100010000?1000000[01]1000000010000010101101??00011100100000101 1
00000000000010101000010000201??1000010000001001001101000000101 0001?11101100010001001000110000 1
100110[01]000?10000010001?00100110[01]00000000000100021100210000101011010001100 0?0?[01]0

Dubreuillosaurus

01???100?0010101?000001001[12]0001??0??0??0??002??001?0????1011?001011?000000??????????10??
2011100?0??110010??0??????010?0110111??201???????0000000?0100000100?1001?????????0?????????0?00??0?
1??????1???????1??000?10????0110?????0??
?????????????11????0?0???????????1?01????????????????1??????1?

Duriavenator

00???100??0101011000?0?0?120?1???0??
????????????????????????110101????????????0000010?0??0?00100??
??
??12

Elaphrosaurus

??
??0110[12]????????00100000?011000001?000000000
2111?0000011??010???????0???11?01110100001000??????????????????000111000000?111111100?[12]??????0?
0?00001100110?00110??00010000210??21?0??????111?001???1??????

Eocarcharia

?????????0000001110010100311111 1?0??????????????????????01?00201111???1??????????????????????
???1000010?01?????10???
??
???10

Eustreptospondylus

?1???20000011?01100000010120?1???0???????100200001??????10?10001?1??00000???0111100010??10
01000100?11000012??????????00110111????????????00000?0?01000001001100 1???011010010001101100000100
11000101?100??1?0000????1????????111?0???0121210?00??????????????????????00110101100011?011300?2?1
?000001010100?1111110000110000100002111101?110102111??1001??000??1?

Fukuiraptor

???????????????????0?0?0??
???????????????????????0????????????????1000010?0110????00110?[12]????????1??????????0????0????????11??
????????????????????????????12001211101?001?1010??????????????10?????10????????????????????????????????
21012101?1100011??01??12????01112121??012?1???0??????

Giganotosaurus

02???000??0?00?11?100001031101???10?1110100021110????????2021211??11211??0??1?00020?110121
???00100?0011211?1??????111??201010?????1?1111110?0020000??00010011012?01?0??111001?01?1?00?100?1

1001?21?2?????2?00000???1??0?10??0???01?0?????????????????????????????????00?11010010211110110201201?
12002011110002201 1101??1?0???1001?31221010?10???????????????????00

Irritator

?????2????02?0???????01100200?100010??0000010?201110010000??00011?11000????0?0???11?10??10?
10010001021000?101?10?????0????????????10?11?0???1101?1-
??????1???
??1??0

Leshansaurus

???????????1?1?1?001??1?0????1????????????????????????????????????10000?????????????0000000100
21001??00????0??????????????????????????????00???0????????[01]0?1100?1?0010??0110000121?0000??0????00
0001100???1????????????0?0??????0110?0111011?[12]0????????0?
?0??????[01]????100001???1?10???211110?0??????????????????0????0

Lourinhanosaurus

??
???1100?????????0??????1?1?000?000?0?00???1?1?0?
?1100000?????0?111??00?01010?1011111011?2?????1???0?001100??1
1011101?0110?????01?21?2101?????????????????????????

Magnosaurus

?????????????????????????????????0??
??????????????????????110?11????????????0000000?0????????0???0??????????????????????????????0??????????
???00???0???01??1?????????????????0??0?????????1???
????0?110?001000?21?????0?11?????????????????????

Majungasaurus

02101000100000010000000100100000011120?10020000?10120000002020201 2?11110110110000100 0101
00?00000010?01101?100000001010110010100110010001001010000000100001000110210011001001100012 1100
0000101000000 1021?1111001201?01000011?1011?10?01110200001 0??????0????????????0??0011100000001111
111??????0???1?????????0????100110?10001010021021111111021111?0001??1101?00

Mapusaurus

??????0???00000111100001001101?1010111101000211 10??1111102?2121121????????[02]01?000?0????
????????????????????????????00201010?01001??????1000020000?0???10011012???01???1??0?0010??0001??01
?00102012?????210000?????????1?11012?0????1???10?10???????0????1????????000???0?0?102?1110110[12]???
??????0?011?100022??1101111?00?1100??31221010110??2111012101??0010?10

Marshosaurus

000??0000000010010001102011001?000010?000??????????0?1001?10000????1000000?10?0?01?010002
0000001001?0?00?1?0????????????10001??0???1?011??001001000100000100110 01?0111110001000012110 00000
0012001101?1??????????????10???????0?1????????????1????????????????????????00?110100101011?011001020
1?0000?00111000?????1??????????????????21???????0?????????????????10

Masiakasaurus

0?????0???0010010000010000100???0?????????10???????????0??20101?10?????????0?001??00????000
?0001??00???0100?????????11101000011-
0??00?000100000000???0020001102?0011001001100011?0000001010000000020111100010 11?0100001?010111
110011101000 01???????????????????001001?100000011?1111111201000 0100 1001??1001101001102100 01010
02102?1111112021111?0011??1101?10

Megalosaurus

?????????0000?01101100?001?0?1?000?????????????????1?111??????????????????????????????????????
?????????????????????????000101?????0?01100001 0010?01??00?100???01????????0??????1?????0?1????1000201
1100001?0000???010?????01110?000001212110?000010000?????????????001010101101011 10113?00[12]??????
0?10??1?0011101100001?000010000221??0?0?1????1????1001??00???00

Megaraptor

???
??11012????????11?0100121?0????????1???????????
???110?0???????1?111011?0121????????????0101011?2?01011111110??????????????????????2?????????????????
???????????????????????????????????????2?11?????????

Metriacanthosaurus

???
???1001?????????0??0??1?1?00011?00?00012111????
?1?000?0???1????1???00??10100101111?011??012?1?0?00?101?01?11
1??110?11110001??0???21?????????????????????????????

Monolophosaurus 001?1000?00100??????000003000?100011101300100?-
0012[01]11000110010?11110001?021000000??1000100000010?????0????01?1??????001000010020100?0???00?0
010?0??0000101110010?0010010010?0010110000000?10000101?10?0?1?000?00?010??????????????????????????
?????????????????????0011000101010111011?00?[12]0100???0001000?0??????????????????????????????????????
???????????????

Neovenator

00???1000001000101010000031001???10110110???
????????????????1?????????110000????????????1000010001?0200100110 12?1?????1111010012110001000 11010
10110?0???2100001???1?10?10?1011?0120????????????????????????????????01?110100102211101102012 01?120
00001?1011020?110111100011100113122 1010?10???1????210111?010?10

Ornitholestes

000??000?00000??????000001100?1?0000100000 1010?0012?11000001000?1??10001?020?00??0??1000?
00???0????0??0??????????????00000?0??0?0?0?0100111?000000??00001001100[12]?????????0100101?0?000?00
0100000000 10100??1?00010??010??11??????????0011110000000 0???01??11??111??1??00?000101112111 1011020
????????0100100000 21??211??0100001???????2??0?0???????????2001??0??????

Piatnitzkysaurus

??????????000?0100001002 0110?1???0??????????????????????????????????1???0?????????????10?0100000

010?1101000?10???????????2010?1????????????0010010?01?????00001001?001111000100001211000010001200
?1011100001?000?0??010?????01011?112001211101100??100?1??????????????001?10100101011?011?0002?100
00000011000011?11100001000001000022121010?10???1????1001??0????10

Piveteausaurus

??011?000?????????????1000?0?1002
100??1002?110???
???
???

Poekilopleuron

???
???
100?0??1?????111??????????012??10110001000001????0?11????0??
????????????10???2????0?01111021?10????????000???

Proceratosaurus

000??000?00100??????000003100?1??0????????0???????201100000????????????1??20??0??0???????????
?????????0?????????????00[02]00?0??0???00??1?1?110000?0??0000000??????????????????????????????????????
???
???1?

Saurophaganax

???0??????????????????????0????????????????
??1100?1??????????0?001?1?000?0??0??????11?????
???00??0????????10??????????012?0101100????????????????????00001?1010010????????20?[12]01?1?00??01???
??210?110??01???011001?21??????????????????00??????????

Shaochilong

??????????000??11?10100100110111?00???1???????????????????????????1?011?????0?00020011???1?00
00100?0011??120?????????????????????????????1???
???
???00

Shidaisaurus

???0??11000?????????????110?0???????
???0??0??0?00???????????0??0110000???????1????001?00????01211010
0001?00???????0???00??0?1??1???111011020?[12]0??00??20??0?
?0??

Siamotyrannus

???
???0[12]?????????????????????????????0???110100
0?0?000????010???002010100102?11101122012110000??101101?
??

Sinosaurus

?0?0?000?0010???????000203??0?1??0??101300100??000?00?00?01?000???1??0?????1?0??????????????
???????????????????????????00??0?00???1?????????0??0?0??????10?0100[12]?1?00??00010000?001000000100?0
00001??000?1000020??01?010??010001?00011211100000010001???1010111?000000?0101?0000111011?[01]00
2010000?2000101110011100001010 00?1000022001010?0????0???110011100??110

Sinraptor_dongi

000010000000000000000100031111110001101000002110000111000100 01001?110000112001000200110
110001001001001120100111?1111100000011002001111100000 0010?0?10000100110011011000100100001211 00
0110001100121101 0?0?1?000?000010???1?01011100??????????????????????2???????11?000201010010101 11011
2201211100002101101011101110111100001100002212101011010211101210111 0010110

Sinraptor_hepingensis

00001000000000??????010003111?1?00011011000021100001110001000 1001?110000112001000???11?1
2000?00?????01??????1?1??????0000001?00?001???1?000?00?000?10000100110011011000100100001211000110
001?001211?1100011000100?0101?11001011100?0????????????????????????????????000010100101?1110112[12
]0021110100210110?10110?1101111?00??

Spinosaurus

110?1201011210????0??0000020??1??0?????0???
???????????????????????00110?0??011????????010110111-
01311?101100[12]??????????1000??21?0100?00???0?02010?00????000?0??0???????????????????????????????????
???
?2?

Streptospondylus

??
???1100[12]?????????0??0?01??1?0000010?1?0???010?
??????000?0??0?0???0??1?000????????0??
????????01000?01????2???????110102111???????????????

Suchomimus

111102010112101?0?00000100200?1??0?????0???????????????????????????1???????????110110?????????
??????????????????????????110??????1????????010010110?013001011100[12]???????100?0001121?11101000110
00201?10?0???00000???1001?0001011?010001211110001001101 0????????????100011?010?1000111011000?[12]
?0?00?000011?00?11??110100110100100??41211010?102021??0?1??????????10

S._zigongensis

??
??00?0??1?????00?1100[12]?????0010??000012110001000?1?00?011
?1??0??000?100?????????01011?0???0110210010000 10001??2?00111111??00001010?10??1110110101[12]0?100
?020011011111?1??0??110?000?00??211[12]10?0??010???????????????????

Torvosaurus_tanneri

00???100?0010??110110001020000??00????00?101200001?011111011000?[01]??????????001111?01???

?????????????????????????????????010?0?????????????00000100010010010011011010210010010001121100001100110001011100001110000??010?101001110000000121211000000100?0[12]???00110110100000101011010111011 3000[12]?100000200101000 1110?10000110000100002121101011010 21110?1001??0????11

Torvosaurus_gurneyi

???????????100011010000102000000?0??0?00010001?????20???1??01

Tyrannotitan

??????????????????????????????????1?????????????????1111??20?010?0??????????1??0?20?0????????01101?1???????1???1001?1?00??00?1?001?2?1???????000??0??????????11????01???0????0?201?????20?????0?220?1?01111?0?01???????2??????????????????????010???

Xuanhanosaurus

??1100?????????0??????1??????000?0??00??11???????1???????????????0?1???1?200?2111?01000010001102?001101?100??

Yangchuanosaurus

0000?000000000??????000003111?1?0001101100002?1000011100010?010?1?1??0?0?120???0?0?????????????????????????0111???1??00000?1?00???1???1?0???00?0?0??00001001100[12]?0110001?01000012110001000?1?000111010000?0000100?010???000000100101?111011010120?100?020011?11111??0101?110????100??211??0101101021??0??????????????

CV00214

??1100[12]?0110001??1000012110001000???000[12]11??0?????00?1?????????????????????012010?0?00101000??0??000???????000?0010?10?????0110[12]0?[12]0??00?0?001???1?[12]1?10111?110?00???0???1???0?0?1???????01???????0?0???

;

proc/;
